# The dependence of Rev-mediated nuclear export of EIAV mRNA on Rev multimerization is modulated by mRNA length

**DOI:** 10.1128/mbio.00841-26

**Published:** 2026-10-09

**Authors:** Weiwei Ma, Mengmeng Zhang, Xiangmin Zhang, Liuke Sun, Jiwei Li, Yang Zhao, Xiaohua Ma, Weiguo Zhang, Bowen Bai, Xue-Feng Wang, Xiaojun Wang

**Affiliations:** 1 State Key Laboratory of Animal Disease Control and Prevention, Harbin Veterinary Research Institute, Chinese Academy of Agricultural Sciences 687216, Harbin, China; 2 Institute of Western Agriculture, Chinese Academy of Agricultural Sciences 838119, Changji, China; 3 China-Kazakhstan Joint Laboratory for Herbivorous Animal Disease Research, Harbin Veterinary Research Institute, Chinese Academy of Agricultural Sciences 111613, Harbin, China; The University of North Carolina at Chapel Hill School of Medicine, Chapel Hill, North Carolina, USA

**Keywords:** lentivirus, EIAV, Rev, multimerization, nuclear export of mRNA

## Abstract

All lentiviruses encode a regulatory protein, Rev, which mediates nuclear export of viral mRNAs and is required for viral replication, and multimerization of Rev is thought to be critical for its function. The lentivirus equine infectious anemia virus (EIAV) Rev is known to mediate the nuclear export of long-length, unspliced, or incompletely spliced viral mRNA encoding the viral structural proteins (Gag, Pol, and Env), like Rev from other lentiviruses. However, EIAV Rev also mediates the nuclear export of a short-length, fully spliced viral mRNA species encoding the functionally unknown protein Mat. Here, we found that disruption of Rev multimerization impaired Rev activity in mediating nuclear export and expression of *gag-pol* mRNA, but not in mediating nuclear export and expression of *mat* mRNA. Deficiency of Rev multimerization leads to lower viral replication. Further investigation revealed that the activity of multimerization-deficient Revs in mediating nuclear export and expression of long-length EIAV or HIV-1 mRNAs was significantly lower than that of wild-type Rev, while this difference was significantly reduced or absent between the two in mediating nuclear export and expression of short-length EIAV or HIV-1 mRNAs. This suggests that the differential dependence of the nuclear export of *gag-pol* mRNA and *mat* mRNA on Rev multimerization is modulated by the lengths of the mRNA species. Our findings suggest that when targeting short mRNAs, EIAV Rev appears to be able to perform nuclear export activity in a non-multimerized state.

**IMPORTANCE:**

Multimerization of lentiviral Rev is thought to be essential for its function, which is to mediate nuclear export of viral mRNA, a necessary step in lentiviral replication. In equine infectious anemia virus (EIAV), the nuclear export of unspliced viral mRNAs encoding structural proteins (Gag/Pol) is dependent on Rev. Moreover, our recent studies have demonstrated that the nuclear export of a fully spliced mRNA species, *mat*, encoding a small protein (Mat) of unknown function, is also Rev-dependent. In this study, we observed that the expression and nuclear export of EIAV *gag-pol* mRNA were strictly dependent on Rev multimerization, whereas those of *mat* mRNA were not. Interestingly, we also determined that the dependence of Rev-mediated nuclear export of EIAV mRNA on Rev multimerization is strongly associated with lengths of these mRNA species. Our data imply that EIAV Rev is able to exert nuclear export activity in a non-multimerized state.

## INTRODUCTION

All lentiviruses, including human immunodeficiency virus type 1 (HIV-1), encode an essential regulatory protein, Rev (regulator of expression of viral proteins). Rev is a nucleocytoplasmic shuttle protein that facilitates the nuclear export of unspliced or incompletely spliced viral mRNA to generate viral proteins and genomic RNA for replication (1–7). Rev-mediated viral mRNA export is an essential step in lentiviral replication and is a complex process (8, 9). HIV-1 Rev first binds a highly structured RNA sequence (termed the Rev-responsive element, RRE) located in the Env-coding region in the intron-containing viral mRNAs (10). The Rev-RRE complex subsequently hijacks the cellular export factors (such as chromosome region maintenance 1, CRM1) to export the viral mRNA from the nucleus of the host cell to the cytoplasm (11, 12).

Rev multimerization is required for Rev function and, thus, for lentiviral replication (13–15); however, the mechanism underlying this is not well understood. Currently, two models are used to explain Rev multimerization (15). In the first model, one Rev molecule initially binds to the RRE, after which additional Rev molecules bind sequentially to the RRE via the Rev-Rev interaction, suggesting that Rev multimerization occurs after binding of the first molecule to the RRE and implying that the multimerization of Rev is dependent on the RRE (1, 16–19). In the second model, Rev multimerization occurs before binding to the RRE, and preformed multimers of Rev then bind directly to the RRE. This model is supported by the observation that Rev molecules can self-associate in the absence of RNA, suggesting that Rev multimerization is independent of the RRE (13, 14, 20–23). However, whichever model is correct, multimerization is nevertheless essential for Rev function because binding of a single Rev molecule to the RRE is not sufficient to activate mRNA export (13, 14) and disruption of Rev multimerization significantly blocks viral replication (24, 25).

Equine infectious anemia virus (EIAV) is a macrophage-tropic lentivirus that infects only equids. EIAV and HIV-1 share many similarities in viral genome organization, molecular regulation of viral replication, life cycles, and host-virus interaction (26, 27). However, although Rev proteins from all lentiviruses are highly functionally homologous, EIAV Rev has very few sequence similarities with other lentivirus Rev proteins (28). Certain important functional domains of EIAV Rev have been identified, including the nuclear localization signal (NLS), RNA-binding domain (RBD), nuclear export signal (NES), and multimerization domain (28–30). Umunnakwe et al. found that a coiled-coil motif located in the central region of the EIAV Rev mediates its multimerization (31). Our lab recently discovered that, in addition to mediating the expression of the EIAV structural proteins (Gag and Pol), EIAV Rev also mediates the expression of Mat, a viral protein of unknown function derived from EIAV that is expressed by a novel fully spliced EIAV transcript (32). The first exon of *mat* mRNA, a 393 nt fragment within the Gag-coding region, can function as RRE by binding Rev and mediating the nuclear export and expression of *mat* mRNA. To distinguish it from the previously identified 555 nt RRE located in the Env-coding region, we herein designate the previously identified element as RRE1 and the first exon of *mat* mRNA as RRE2 (32–34). We also found that the nuclear export activities of Rev-RRE1 and Rev-RRE2 are dependent on different amino acid sites and regions of Rev, implying that the molecular mechanisms underlying EIAV Rev-mediated expression of Gag-Pol and Mat are different (32).

Lentiviruses, including EIAV, have high mutation rates, resulting in viral populations with multiple, diverse variants known as viral quasispecies (35–39). Previous studies have found that the EIAV *rev* sequence is highly variable both *in vivo* and *in vitro*, and that changes in the Rev phenotype coincide with changes in the clinical stage of disease (40–43). In the case of HIV-1, the Rev-attenuated variants have also been found in the asymptomatic stage (44–47). Importantly, the attenuation of Rev activity can reduce the expression of viral structural proteins (Gag, Pol, Env), which contributes to lentivirus evasion of host immune clearance and establishment of lentiviral latent infection (47). In this study, we found a natural glutamate (E)-to-lysine (K) mutation (E109K) in Rev from the bone marrow of EIAV-infected horses that impaired Rev multimerization and consequently led to disruption of Rev-mediated Gag-Pol expression, but not Rev-mediated Mat expression. Our data further show that differences in the lengths of the *gag-pol* and *mat* mRNAs affect the differential tolerance of their nuclear exports to changes in Rev multimerization. Overall, these results suggest that EIAV Rev is able to exert nuclear export activity in a non-multimerized state.

## RESULTS

### A natural E109K mutation in EIAV Rev attenuates its activity in mediating Gag-Pol expression without affecting mediation of Mat expression

**Fig 1:**
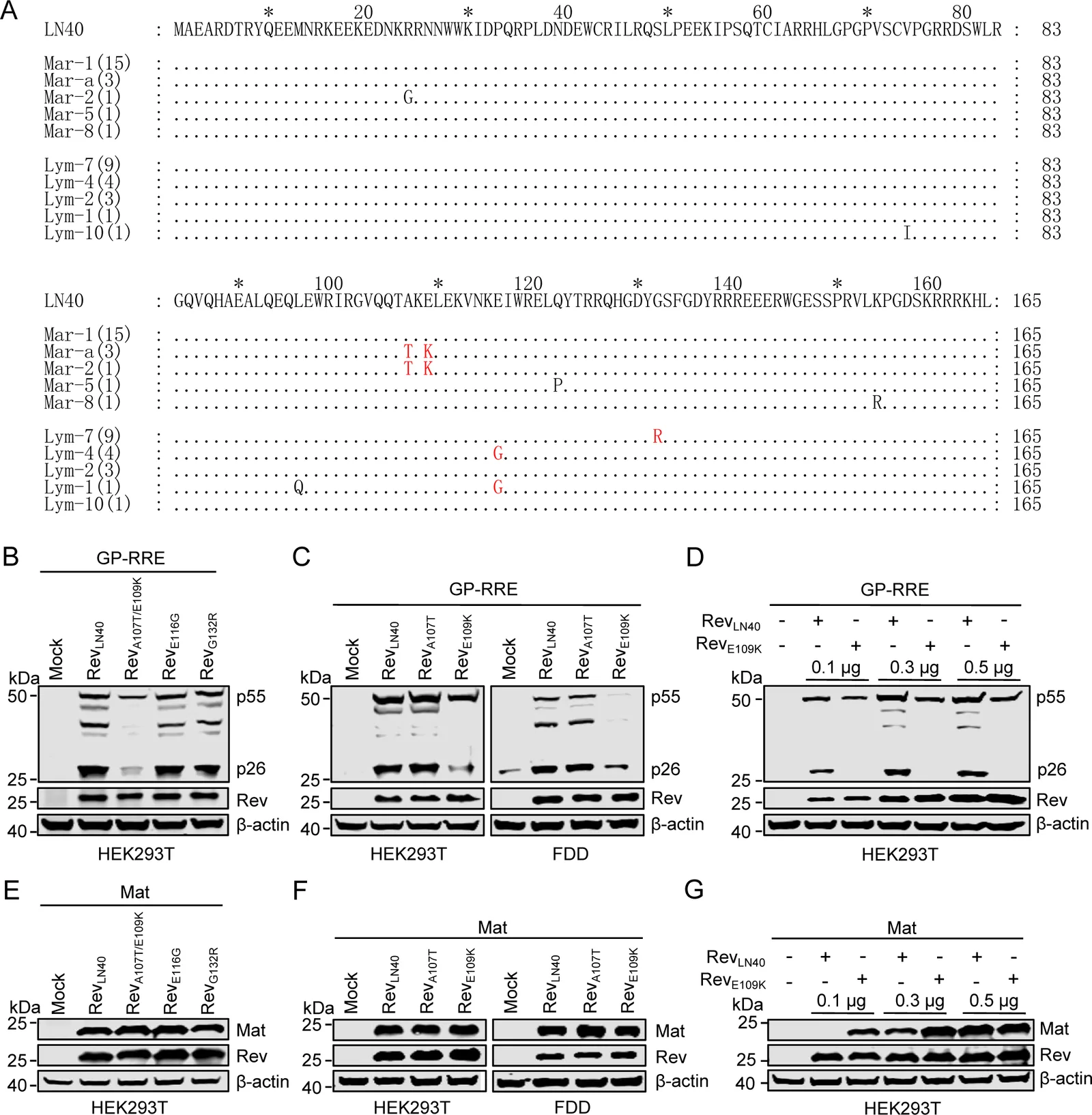
An E109K mutation in EIAV Rev attenuates its activity in mediating Gag-Pol expression without affecting its activity in mediating Mat expression. (**A**) The multiple sequence alignment of the amino acids encoded by the *rev* mRNA isolated from bone marrow (Mar) and lymph nodes (Lym) of an EIAV_LN40_-infected horse. The Rev sequences were aligned to the consensus sequence of EIAV_LN40_, shown at the top. Only the amino acid residues that are different from the consensus sequence of EIAV_LN40_ are indicated, and dots indicate residues identical to the consensus sequence. For each sample, the different sequences are listed on the left, with the name of the sequence and the number of clones with a given sequence in parentheses. Red letters indicate functional tests of the mutation in the following experiments. Asterisks above the sequence mark every 10‑amino‑acid residue position. (**B and C**) HEK293T cells or FDD cells were cotransfected with a Rev-dependent EIAV Gag-Pol expression construct (pGP-RRE) and either a series of Rev expression plasmids as indicated (**B and C**), or increasing amounts (0.1–0.5 μg) of Rev_LN40_ or Rev_E109K_ expression plasmids (**D**), and an empty vector VR1012 was cotransfected as a negative control, Mock. The cells were collected at 48 hpt and were lysed for the detection of Gag and Rev expression using anti-p26 antibody and anti-FLAG antibody, respectively. To ensure uniform blotting and even loading of the gel, β-actin expression was detected using a rabbit anti-actin antibody. (**E–G**) HEK293T cells or FDD cells were cotransfected with a Mat construct and either a series of Rev expression plasmids as indicated (**E and F**) or increasing amounts (0.1–0.5 μg) of Rev_LN40_ or Rev_E109K_ expression plasmids (**G**), and an empty vector VR1012 was cotransfected as a negative control, Mock. The cells were collected at 48 hpt and were lysed for the detection of Mat and Rev expression using anti-hemagglutinin (HA) antibody and anti-FLAG antibody, respectively. All the experiments were performed three times, and a representative result is shown.

We used RT-PCR to amplify the complete *rev* gene from the bone marrow and lymph glands of an EIAV-infected horse inoculated with a virulent EIAV strain (EIAV_LN40_) and that died 18 days post-inoculation (48). A total of 18 and 21 clones were obtained from bone marrow and lymph glands, respectively, and the deduced Rev amino acid sequences were aligned with the EIAV_LN40_ consensus reference. A total of 9 amino acid variations were observed in all Rev sequences. Five of these mutations were found only in a single clone (such mutations were considered to be random and were not subject to subsequent functional studies). However, a further four mutations (A107T, E109K, E116G, and G132R) were found in more than two clones derived from three Rev variants. (Fig. 1A).

To assess whether the four mutants were able to affect the function of Rev, we then constructed four Rev expression vectors, including wild-type Rev (Rev_LN40_) and three Rev variants containing the four mutations mentioned above (Rev_A107T/E109K_, Rev_E116G_, and Rev_G132R_). A Rev-dependent EIAV Gag-Pol expression vector, pGP-RRE, was cotransfected with Rev_LN40_ or the other Rev mutants in HEK293T cells. Gag protein expression in cells was subsequently measured using western blotting (WB). We detected less Gag protein in the presence of Rev_A107T/E109K_ compared to the presence of Rev_LN40_, while there was no significant change in Gag protein expression in the presence of the other Rev mutants (Fig. 1B). Next, single-site mutations were generated to determine whether it was the A107T or E109K mutation that interfered with the activity of Rev in mediating EIAV Gag-Pol expression. pGP-RRE was cotransfected with a vector expressing either Rev_A107T_ or Rev_E109K_ into HEK293T cells or fetal donkey dermal (FDD) cells (which are permissive for EIAV) (49). The WB assay showed that Rev_A107T_ was able to mediate the expression of Gag-Pol like Rev_LN40_, while Rev_E109K_ dramatically attenuated this activity (Fig. 1C and D). These results suggest that the E109K mutation attenuates Rev activity in mediating EIAV Gag-Pol protein expression.

In addition to mediating EIAV Gag-Pol expression, Rev mediates expression of Mat, another EIAV accessory protein, by specifically binding to *mat* mRNA to promote its nuclear export (32). To investigate whether the four mutations were able to affect the ability of Rev to mediate Mat expression, the Mat expression vector was cotransfected with Rev_LN40_ or each of the three Rev mutants separately in HEK293T cells, and Mat expression was then analyzed using WB. Surprisingly, the three Rev mutants were able to mediate Mat expression to the same extent as the wild-type Rev_LN40_ (Fig. 1E). We subsequently also confirmed in both HEK293T cells and FDD cells that neither the A107T nor the E109K mutant affected the mediation of Mat expression by Rev (Fig. 1F). Interestingly, a Rev dose-dependent experiment showed that at low doses of Rev (0.1 and 0.3 μg), Rev_E109K_ was significantly more effective than Rev_LN40_ in mediating Mat expression. However, this difference was abolished at a high dose of Rev (0.5 μg), where both variants showed comparable efficacy in mediating Mat expression (Fig. 1G).

Together, these data demonstrated that the E109K mutation in EIAV Rev attenuates its activity in mediating Gag-Pol expression, but not Rev-mediated Mat expression.

### The presence of lysine (K), but not of other amino acids, at Rev position 109 specifically disrupts its activity in mediating Gag-Pol expression

**Fig 2:**
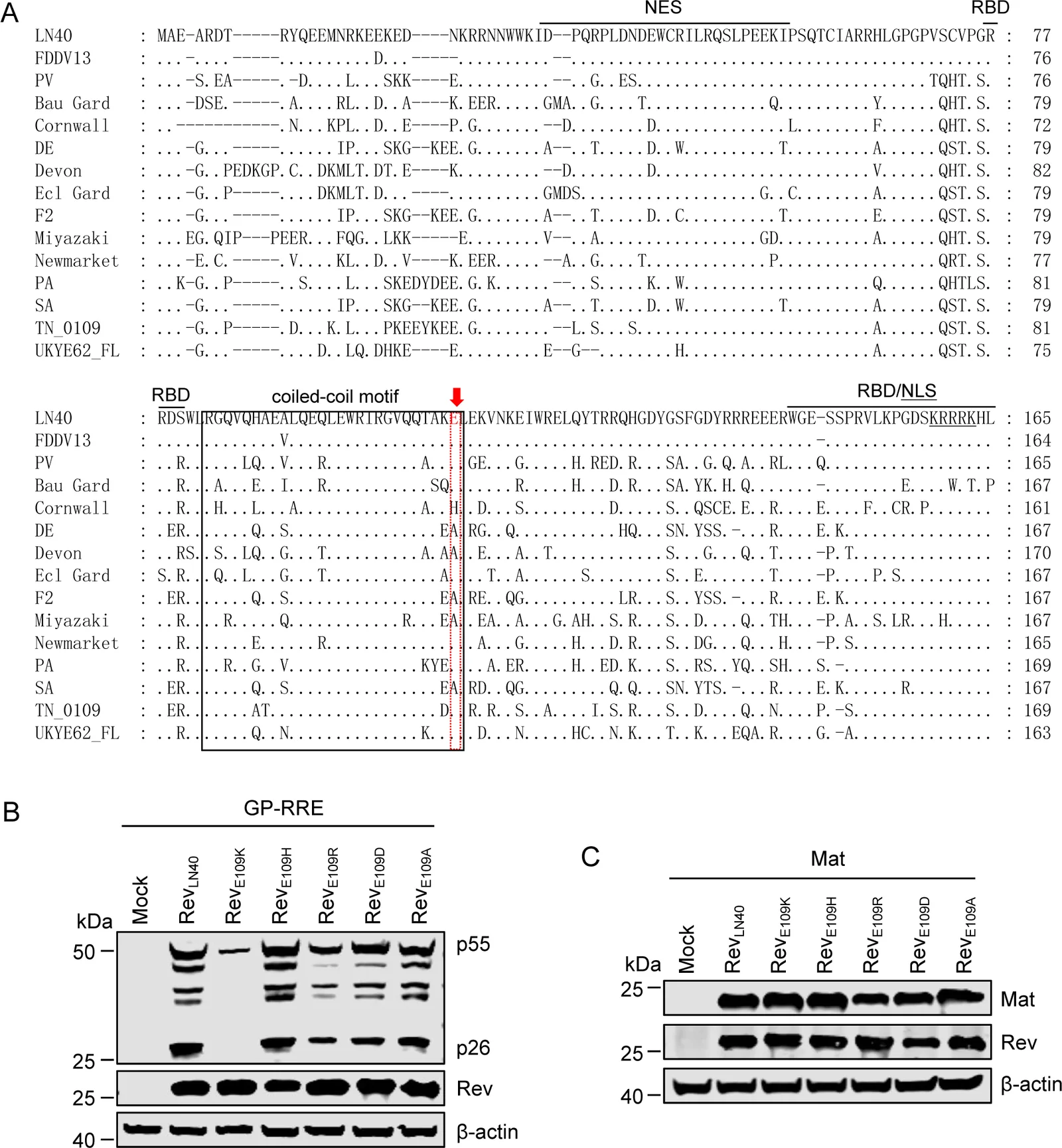
Identifying variability at amino acid position 109 of Rev protein among EIAV strains. (**A**) Alignment of the Rev amino acid sequence from different strains of EIAV. All Rev sequences were aligned to EIAV_LN40_, shown at the top. Only the amino acid residues that were different from the Rev consensus sequence of EIAV_LN40_ are indicated, and dots indicate residues identical to the consensus sequence. The black box indicates the previously identified predicted coiled-coil motif responsible for EIAV Rev multimerization. The red arrow and red dashed box indicate the variability of amino acids at position 109 (corresponding to EIAV_LN40_) of the Rev amino acid sequence. NES, nuclear export signal; RBD, RNA-binding domain; NLS, nuclear localization signal. (**B and C**) HEK293T cells were cotransfected with the pGP-RRE construct (**B**) or Mat construct (**C**) and a series of Rev expression plasmids as indicated, or an empty vector VR1012 (as a negative control, Mock). The cells were collected at 48 hpt and were lysed for the detection of Gag, Mat, and Rev expression using anti-p26 antibody, anti-HA antibody, and anti-FLAG antibody, respectively. β-Actin expression was detected using a rabbit anti-actin antibody. All the experiments were performed three times, and a representative result is shown.

To investigate whether the E109K mutation occurs in other EIAV strains, we performed a sequence alignment of the Rev protein sequences from several different EIAV strains (LN40, GU385365; FDDV13, GU385361; PV, AF005148; Bau Gard, MK593462; Cornwall, MH580898; DE, KM247554; Devon, MH580897; Ecl Gard, MK593463; F2, JX480631; Miyazaki, JX003263; Newmarket, MH580896; PA, GQ855756; SA, KM247555; TN_0109, MH820166; UKYE62_FL, MH820163). As shown in Fig. 2A, at Rev residue 109 (numbered according to the EIAV_LN40_ strain sequence), five EIAV strains had an alanine (A), one strain had a histidine (H), and the others all had a glutamic acid (E). In Rev_LN40_, the amino acid at position 109 is E, an acidic amino acid. To further confirm the effect of the amino acid property of Rev at position 109 on its nuclear export activity, we mutated the amino acid at position 109 of Rev_LN40_ to H (a weakly basic heterocyclic amino acid), R (a basic amino acid), D (an acidic amino acid), and A (a neutral amino acid). This resulted in four Rev mutants, namely, Rev_E109H_, Rev_E109R_, Rev_E109D_, and Rev_E109A._ The pGP-RRE or Mat expression vectors were separately cotransfected into HEK293T cells with each Rev mutant expression vector. To serve as positive and negative controls, both Rev_LN40_ and Rev_E109K_ were included. The results showed that the activity of Rev in mediating Gag-Pol expression was dramatically decreased when position 109 was K. A moderate decrease in activity was observed when the position was R, while it was unaffected when the position was one of the other amino acid types (Fig. 2B). Moreover, the activity of Rev in mediating Mat expression was not affected by the amino acid type at position 109 (Fig. 2C). Therefore, the reduced activity of Rev in mediating Gag-Pol expression caused by the E109K mutation is amino acid specific.

### The E109K mutation in EIAV Rev attenuates its ability to mediate nuclear export of *gag-pol* mRNA by disrupting Rev multimerization

**Fig 3:**
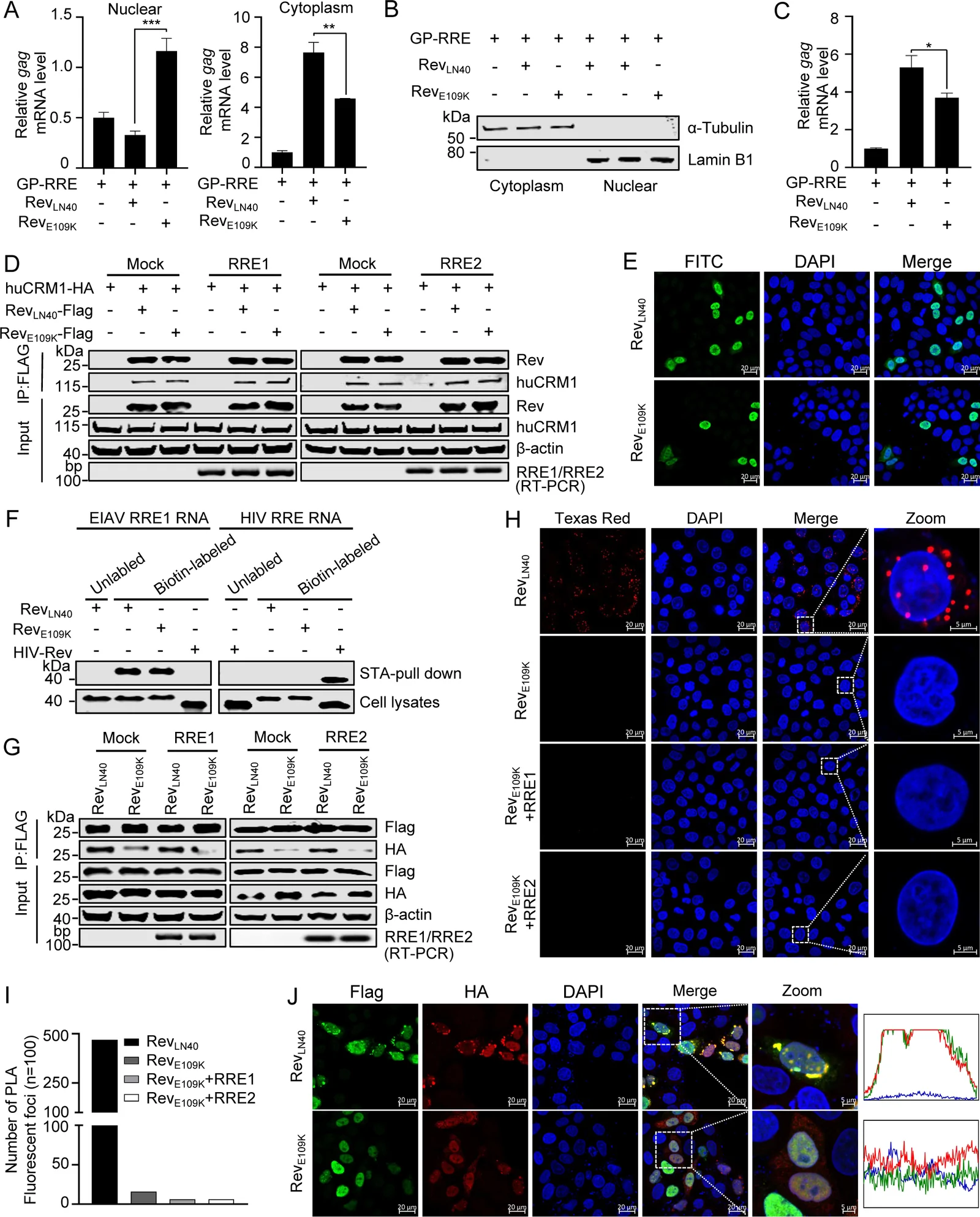
The E109K mutation disrupts Rev multimerization without affecting other aspects of its function. (**A–C**) Rev-mediated nuclear export of *gag-pol* mRNA attenuated by the E109K mutation. HEK293T cells were cotransfected with the pGP-RRE construct and Rev_LN40_, Rev_E109K_ expression plasmids, or an empty vector VR1012 (as a negative control, Mock). The nuclear and cytoplasmic RNA fractions (**A**) or total RNA (**C**) were extracted at 24 hpt. Levels of *gag-pol* mRNA in cytoplasmic and nuclear fractions or in total mRNA were determined using RT-qPCR and the 2^−ΔΔCT^ method. Glyceraldehyde-3-phosphate dehydrogenase mRNA (GAPDH) (for cytoplasmic and nuclear mRNA fractions) and β-actin mRNA (for total mRNA) were measured as an internal reference. Error bars represent standard deviations of three independent experiments. ***, *P* < 0.001; **, *P* < 0.01; *, *P* < 0.05. WB of Lamin B1 and α-Tubulin was conducted to assess the effectiveness of cell fractionation (**B**). (**D**) The E109K mutation did not affect the interaction of the Rev with chromosome region maintenance 1 (CRM1). HA-tagged huCRM1 expression plasmids and FLAG-tagged Rev_LN40_ or Rev_E109K_ expression plasmids were separately cotransfected with RRE1 or RRE2 expression plasmids into HEK293T cells, and an empty vector VR1012 was cotransfected as a negative control, Mock. At 48 hpt, whole-cell lysates were immunoprecipitated with FLAG beads and then analyzed with WB. Total RNA was extracted, and the levels of *RRE1* or *RRE2* mRNA were determined using RT-PCR. (**E**) Confocal microscopy to determine whether the E109K mutation affected the subcellular localization of Rev. HeLa cells were separately transfected with the Rev_LN40_ or Rev_E109K_ expression plasmids. At 24 hpt, the cells were fixed and stained with an anti-FLAG antibody (green), and the nuclei were stained with DAPI (blue). (**F**) An RNA pull-down assay was performed to test whether the E109K mutation affected the Rev binding to RRE. Cell lysates from HEK293T cells transfected with Rev_LN40_, Rev_E109K_, or HIV Rev expression plasmids were incubated with biotin-labeled or unlabeled RNA transcribed *in vitro*. The biotin-labeled EIAV RRE1 mRNA or HIV-1 RRE was pulled down with streptavidin (STA)-conjugated magnetic beads, followed by WB of copurified proteins using anti-FLAG antibody. Biotin-unlabeled RRE mRNA was used to indicate the specificity of this assay. (**G**) Co-immunoprecipitation experiment to test whether the E109K mutation affected the Rev-Rev interactions. HEK293T cells were transfected with FLAG- and HA-tagged Rev_LN40_ or Rev_E109K_ expression plasmids in combination with RRE1 or RRE2 expression plasmids, and an empty vector pcDNA3.1+ was cotransfected as a negative control, Mock. At 48 hpt, whole-cell lysates were immunoprecipitated with FLAG beads and then analyzed with WB. Total RNA was extracted, and the levels of *RRE1* or *RRE2* mRNA were determined using RT-PCR. (**H**) PLA experiment to test whether the E109K mutation affected the Rev-Rev interactions. HeLa cells were transfected with FLAG- and HA-tagged Rev_E109K_ expression plasmids in combination with RRE1 or RRE2 expression plasmids, and FLAG-tagged and HA-tagged Rev_LN40_ expression plasmids were transfected as a positive control. PLA puncta were detected using confocal microscopy. Nuclei were stained with DAPI (blue). (**I**) Quantitative analysis of PLA foci for Rev_LN40_ or Rev_E109K_. The number of PLA foci per cell was quantified in 100 cells for each condition. (**J**) IFA for co-localization analysis of Rev tagged with FLAG and HA. HeLa cells were transfected with FLAG- and HA-tagged Rev_LN40_ or Rev_E109K_ expression plasmids. At 24 hpt, the cells were fixed and stained with an anti-FLAG antibody (green) and an anti-HA antibody (red), and the nuclei were stained with DAPI (blue). The histogram of fluorescent intensity from the merged image was obtained using ZEISS ZEN Blue software. The colored wavy lines, representing the fluorescence of the corresponding colors, show the colocalization of FLAG- and HA-tagged Rev_LN40_ or Rev_E109K_. These experiments were performed three times (A–E, G, and J) or two times (F, H, and I), and a representative result is shown.

Rev is a nucleoplasmic shuttle protein that promotes viral mRNA nuclear export by binding to RRE in a CRM1-dependent manner (8, 50). To investigate whether the reduced Gag-Pol expression mediated by Rev_E109K_ was due to disruption of its activity in mediating the nuclear export of *gag-pol* mRNA, we isolated nuclear and cytoplasmic mRNA from HEK293T cells transfected with pGP-RRE and vectors expressing either Rev_LN40_ or Rev_E109K_. We then analyzed the levels of *gag-pol* mRNA with quantitative PCR (qPCR). As shown in Fig. 3A and B, the accumulation of *gag-pol* mRNA in the cytoplasm was reduced in the presence of Rev_E109K_ compared to that in the presence of Rev_LN40_. Furthermore, we also found that the total *gag-pol* mRNA was significantly reduced in the presence of Rev_E109K_ compared to that observed in the presence of Rev_LN40_ (Fig. 3C). These results suggested that the E109K mutation impairs Rev-mediated nuclear export of *gag-pol* mRNA, leading to nuclear retention of *gag-pol* mRNA and triggering nuclear degradation, thereby reducing the expression of Gag-Pol and total cellular *gag-pol* mRNA levels mediated by Rev.

The interaction with CRM1, its nuclear localization, the binding of Rev to the RRE, and Rev multimerization are all essential for Rev-mediated nuclear export of lentiviral mRNA. To investigate how Rev-mediated nuclear export of EIAV *gag-pol* mRNA was affected by the E109K mutation, we performed the following series of experiments.

We used co-immunoprecipitation (Co-IP) to test whether the E109K mutation affected the interaction of Rev with CRM1, which is critical for Rev-mediated *gag-pol* mRNA export. HEK293T cells were cotransfected with hemagglutinin (HA)-tagged CRM1 and FLAG-tagged Rev_LN40_ or Rev_E109K_ expression vectors in the presence or absence of RRE1 or RRE2, and the interaction between Rev and CRM1 was subsequently assessed using FLAG antibody-based Co-IP. The results demonstrated that the amounts of CRM1 coprecipitated by Rev_E109K_ and Rev_LN40_ were similar (Fig. 3D). Thus, the E109K mutation did not interfere with the interaction between Rev and CRM1.

Under steady-state conditions, EIAV Rev is mainly localized to the nucleus (51). We therefore performed confocal microscopy to investigate the subcellular localization of Rev_LN40_ or Rev_E109K_. We found that both were mainly localized in the nucleus (Fig. 3E). Thus, the cellular localization of Rev was not affected by the E109K mutation.

RNA pulldown assays were performed using biotin-labeled EIAV RRE1 RNA and streptavidin (STA) magnetic beads to determine whether the E109K mutation affected the binding of Rev to the EIAV RRE1. WB analysis with anti-FLAG antibody showed that similar amounts of Rev_LN40_ or Rev_E109K_ were specifically bound to the EIAV RRE1 mRNA (Fig. 3F). These results indicated that the E109K mutation did not impair the binding of Rev to the EIAV RRE1.

Multimerization is required for Rev to mediate lentiviral Gag-Pol expression (15). To investigate whether the E109K mutation disrupts Rev multimerization, FLAG-tagged Rev_E109K_ expression plasmid was cotransfected with HA-tagged Rev_E109K_ expression plasmid into HEK293T cells in the presence or absence of RRE1 or RRE2, and the Rev self-association was assessed using a Co-IP assay, with FLAG- and HA-tagged Rev_LN40_ as a positive control. We found that the capability of Rev_E109K_ to self-associate was dramatically attenuated compared to that of Rev_LN40_ (Fig. 3G). In addition, a proximity ligation assay (PLA) also showed that the E109K mutation disrupted Rev self-association, regardless of the presence or absence of RRE1 or RRE2 (Fig. 3H and I). Furthermore, immunofluorescence analysis (IFA) demonstrated that clear co-localization of FLAG- and HA-tagged Rev proteins was observed exclusively for the Rev_LN40_, which overlapped in both the cytoplasm and nucleus; by contrast, no detectable co-localization was observed for the Rev_E109K_ mutant (Fig. 3J).

Collectively, the reduced nuclear export of *gag-pol* mRNA by Rev_E109K_ may involve impairment of Rev multimerization rather than nuclear localization of Rev or interaction of Rev with the EIAV RRE or CRM1.

### The E109K mutation in Rev reduces EIAV production and replication *in vitro*

**Fig 4:**
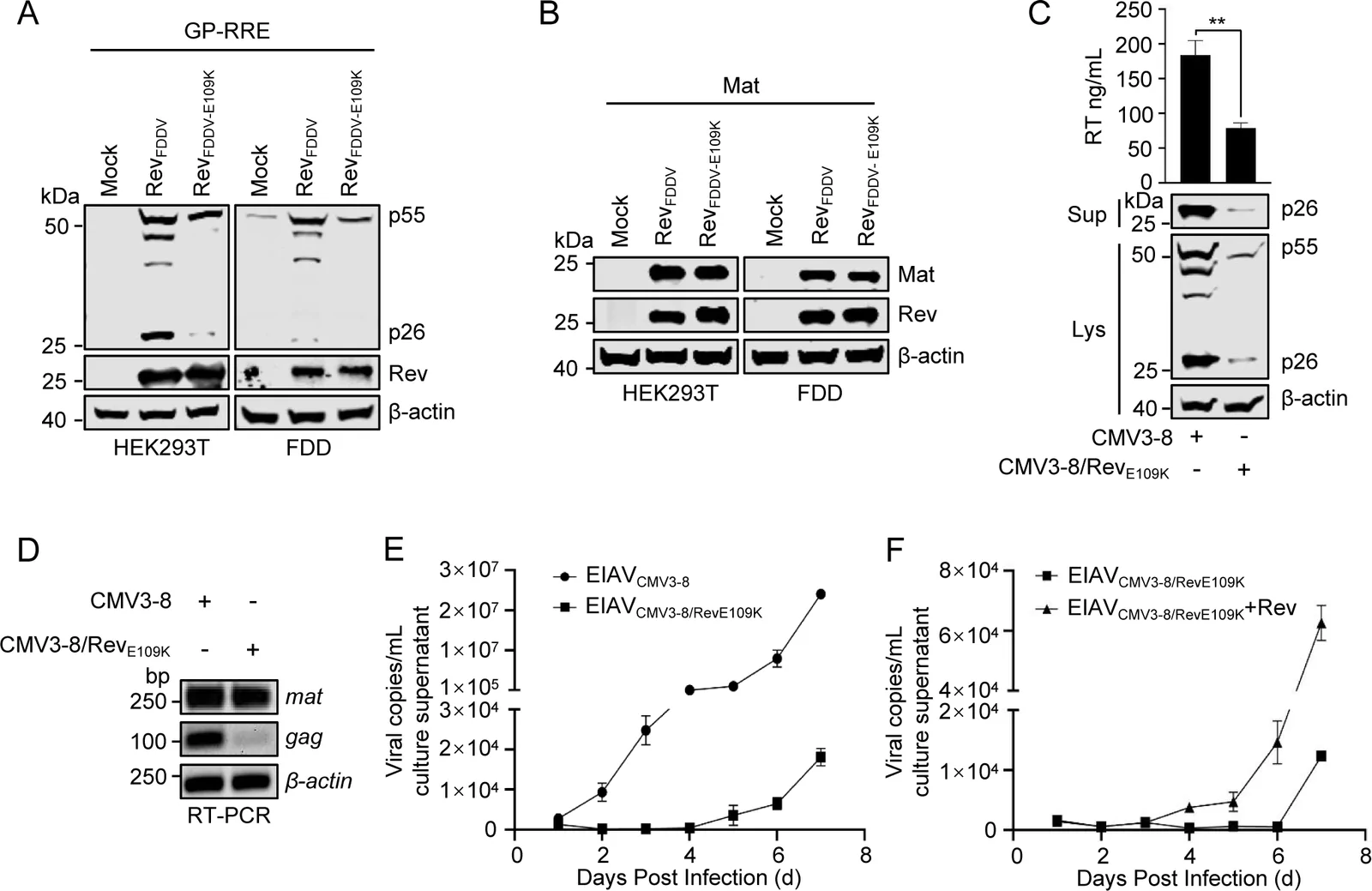
The E109K mutation reduces EIAV production and attenuates EIAV replication *in vitro*. (**A and B**) Investigation of the effect of the E109K mutation on Rev activity in the context of EIAV_FDDV13_ Rev. HEK293T cells or FDD cells were cotransfected with the pGP-RRE construct (**A**) or a Mat construct (**B**) together with Rev_FDDV_ or Rev_FDDV-E109K_ expression plasmids, or an empty vector VR1012 (as a negative control, Mock). The cells were collected at 48 hpt and were lysed for the detection of Gag, Mat, and Rev expression using anti-p26 antibody, anti-HA antibody, and anti-FLAG antibody, respectively. β-actin expression was detected using a rabbit anti-actin antibody. All the experiments were performed three times, and a representative result is shown. (**C**) The E109K mutation reduces EIAV production. HEK293T cells were transfected with equivalent amounts of CMV3-8 or CMV3-8/Rev_E109K_. At 48 hpt, culture supernatants were ultracentrifuged to concentrate the viral particles after removing cell debris. Viral CA protein (p26) in the supernatant and Gag protein in the cell lysates were analyzed by WB using anti-p26 antibody. Virions released from HEK293T cells transfected with CMV3-8 or CMV3-8/Rev_E109K_ were analyzed using a reverse transcriptase (RT) activity assay. The data represent the means ± SD from three independent experiments. Error bars represent standard deviations of three independent experiments. **, *P* < 0.01. (**D**) The E109K mutation in EIAV reduces the production of *gag-pol* mRNA. HEK293T cells were transfected with either CMV3-8 or CMV3-8/Rev_E109K_. Total RNA was extracted at 24 hpt, and the levels of *gag-pol* mRNA and *mat* mRNA in the total RNA were determined using RT-PCR. *β-actin* mRNA served as an internal control for the RT-PCR. (**E**) Growth kinetics of EIAV_CMV3-8_ and EIAV_CMV3-8/RevE109K_. The same amount (RT) of EIAV_CMV3-8_ and EIAV_CMV3-8/RevE109K_ was inoculated into eMDMs, and viral RNA was prepared from the supernatants at the indicated time points after infection and quantified using qPCR. (**F**) Rescue of EIAV_CMV3−8/RevE109K_ replication in eMDMs by *trans* complementation of Rev. Equivalent amounts of EIAV_CMV3−8/RevE109K_ with pseudotyped HIV-1 containing Rev or without Rev (as a specificity control) were inoculated into eMDMs. Viral RNA was prepared from the supernatants at the indicated time points after infection and quantified using qPCR. The results represent the means of three independent infections, and error bars represent standard deviations.

To assess the effect of the E109K mutation in Rev on EIAV production, we generated an EIAV molecular clone with the E109K mutation, CMV3-8/Rev_E109K_. This was based on a full-length EIAV molecular clone, CMV3-8, derived from the EIAV attenuated strain EIAV_FDDV13_ (52). Prior to performing this experiment, we confirmed that the E to K mutation in the context of Rev_FDDV_ also impaired its activity in mediating Gag-Pol expression (Fig. 4A) but did not affect its activity in mediating Mat expression (Fig. 4B). Equivalent amounts of CMV3-8 or CMV3-8/Rev_E109K_ were transfected into HEK293T cells. At 48 hpt, WB was used to measure the levels of EIAV Gag and capsid (p26) in cell lysates and culture supernatants, respectively, and reverse transcriptase (RT) activity was assayed to quantify viral particle production. As shown in Fig. 4C, the expression of Gag in the lysate and the expression of p26 in the culture supernatant of cells transfected with CMV3-8/Rev_E109K_ were significantly decreased compared to cells transfected with CMV3-8. Consistently, the RT activity assay also confirmed that cells transfected with CMV3-8/Rev_E109K_ produced significantly fewer viral particles in the supernatant than cells transfected with CMV3-8. These data showed that the E109K mutation in Rev reduces EIAV production.

To demonstrate that the reduction in EIAV production was due to the absence of EIAV mRNA encoding the viral structural protein Gag-Pol (*gag-pol* mRNA) in these cells, we harvested total RNA from HEK293T cells transfected with CMV3-8 or CMV3-8/Rev_E109K_ and assessed the levels of *gag-pol* mRNA and *mat* mRNA in these transfected cells using RT-PCR. As expected, the level of *gag-pol* mRNA was significantly lower in cells transfected with CMV3-8/Rev_E109K_ than in cells transfected with CMV3-8, while the level of *mat* mRNA did not differ between the two (Fig. 4D). These findings demonstrated in the context of an EIAV infectious molecular clone that the E109K mutation attenuates Rev-mediated expression of Gag-Pol without affecting Rev-mediated expression of Mat. Subsequently, equine macrophages (eMDMs), which are the primary target cells of EIAV, were infected with equal amounts (RT) of either EIAV_CMV3-8_ or EIAV_CMV3-8/RevE109K_, and viral growth kinetics were determined using qPCR. The results showed that the E109K mutation in Rev significantly attenuated EIAV replication *in vitro* (Fig. 4E). However, *trans* expression of Rev_LN40_ in eMDMs via pseudotyped HIV vectors restored the replication of the EIAV_CMV3-8/RevE109K_ (Fig. 4F). This result confirms that the replication defect of EIAV_CMV3-8/RevE109K_ is directly caused by the impaired multimerization function of the Rev_E109K_ mutant.

### The lengths of EIAV mRNAs modulate their nuclear export dependence on Rev multimerization

**Fig 5:**
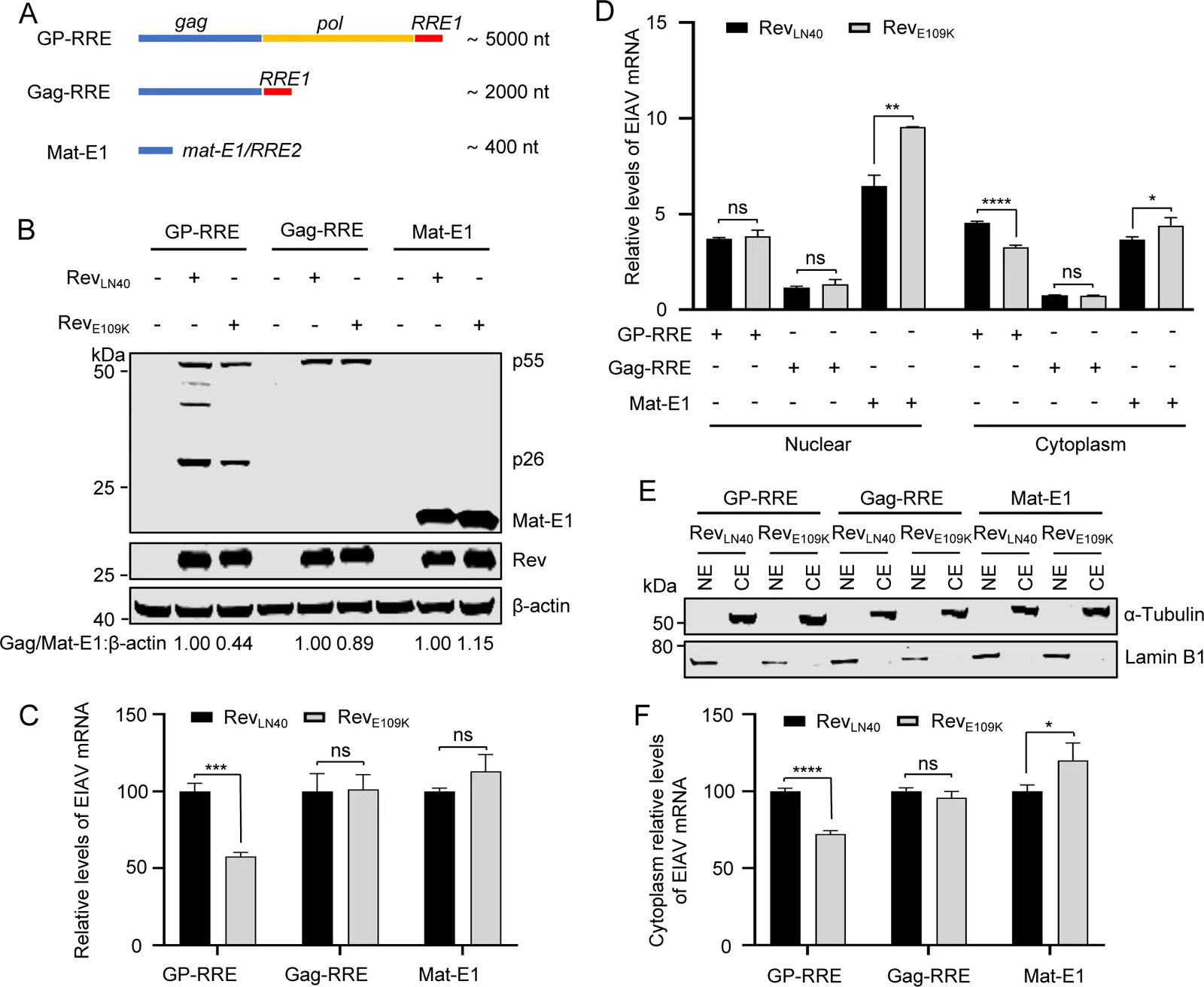
Comparison of the dependence of nuclear export of three EIAV mRNA species with different lengths on Rev_LN40_ and Rev_E109K_. (**A**) Schematic diagram of a series of plasmids transcribed to produce EIAV mRNA species with different lengths. GP-RRE contains EIAV *gag-pol*-coding sequences and an intact Rev responsive element (RRE1) that generates approximately 5,000 nt of EIAV mRNA. Gag-RRE contains EIAV *gag-*coding sequences and an intact RRE1 that generates approximately 2,000 nt of EIAV mRNA. Mat-E1 contains the first exon of the EIAV *mat* gene, which also functions as an RRE (RRE2), producing approximately 400 nt of EIAV mRNA. (**B**) WB analysis of indicated plasmids cotransfected with Rev_LN40_, Rev_E109K_ vector, or an empty vector VR1012 (as a negative control, Mock). Band intensities of Gag and Mat-E1 were determined with Odyssey CLx Image Studio and normalized to β-actin. Values were then normalized to control cells, with the control set as 1. (**C–F**) RT-qPCR analysis was performed to determine the levels of EIAV mRNA in cells transfected with the indicated plasmid pairs. HEK293T cells were cotransfected with the indicated plasmid pairs. Total RNA (**C**) or the nuclear and cytoplasmic RNA fractions (**D**) were extracted at 24 hpt, and levels of EIAV mRNA were determined with RT-qPCR using specific primers. Lamin B1 and α-Tubulin were detected with WB to assess the effectiveness of cell fractionation (**E**). The relative cytoplasmic expression levels of each EIAV mRNA in the presence of Rev_LN40_ or Rev_E109K_ were compared (**F**). The relative level of each EIAV mRNA species in the presence of Rev_LN40_ was normalized to 100. Error bars represent standard deviations from three independent experiments. ****, *P* < 0.0001; ***, *P* < 0.001; **, *P* < 0.01; *, *P* < 0.05; ns, not significant (*P* > 0.05). All the experiments were performed three times, and a representative result is shown.

As shown previously, RNA length is a key determinant of RNA nuclear export (53, 54). In the above experiments, we observed that the Rev_E109K_ with impaired multimerization showed a diminished ability to mediate Gag-Pol expression, while its ability to mediate Mat expression was unimpaired (Fig. 1 and 4). These results may indicate that impairment of Rev multimerization has a different effect on nuclear export of *gag-pol* and *mat* mRNA. To further investigate whether this difference was due to their different lengths, we prepared three constructs that could be transcribed to produce EIAV mRNA species with decreasing sequence length (Fig. 5A). GP-RRE transcribes an EIAV mRNA species of approximately 5,000 nt (called long-length EIAV mRNA) containing the complete *gag-pol* open reading frame (ORF) and intacting Rev responsive element (RRE1). Rev-mediated expression of this mRNA results in EIAV Gag-Pol. Gag-RRE transcribes an EIAV mRNA species of approximately 2,000 nt (called medium-length EIAV mRNA). This mRNA contains the complete EIAV *gag* ORF and an intact RRE1. Rev-mediated expression of this mRNA results in EIAV Gag. Mat-E1 transcribes an EIAV mRNA species of approximately 400 nt (called short-length EIAV mRNA). This mRNA contains the first exon of the *mat* gene overlapping with 393 nt of the N-terminal region of the *gag* gene, which also functions as an RRE (RRE2) and expresses a small protein mediated by Rev (32). WB and qPCR assays showed that there was no difference in the activity of Rev_LN40_ and Rev_E109K_ in mediating the expression of the medium- and short-length mRNAs, except that the activity of Rev_E109K_ was significantly less than that of Rev_LN40_ in mediating the expression of the long-length mRNA (Fig. 5B and C).

We next wanted to test whether there was a difference between the efficiency of Rev_LN40_- or Rev_E109K_-mediated nuclear export of these three EIAV mRNA species of different lengths. We isolated nuclear and cytoplasmic mRNA from HEK293T cells transfected with each of the three constructs and vectors expressing Rev_LN40_ or Rev_E109K_ and analyzed the levels of cytoplasmic and nuclear EIAV mRNA with qPCR (Fig. 5D through F). The results showed that the activity of Rev_E109K_ in mediating the cytoplasmic accumulation of the long mRNA was significantly lower than that of Rev_LN40_, and there was no difference in mediating the accumulation of the medium-length mRNAs, while in mediating the cytoplasmic accumulation of the short-length mRNA, the activity of Rev_E109K_ was slightly higher than that of Rev_LN40_. These results suggest that, compared to Rev_LN40_, the multimerization-deficient Rev_E109K_ is more efficient at mediating the nuclear export and expression of short mRNA than long mRNA. This implies that the dependence of nuclear export of EIAV mRNA on Rev multimerization may be correlated with the length of the mRNA.

### The difference in nuclear export activity of Rev_LN40_ and Rev_E109K_ was independent of the RRE pattern

**Fig 6:**
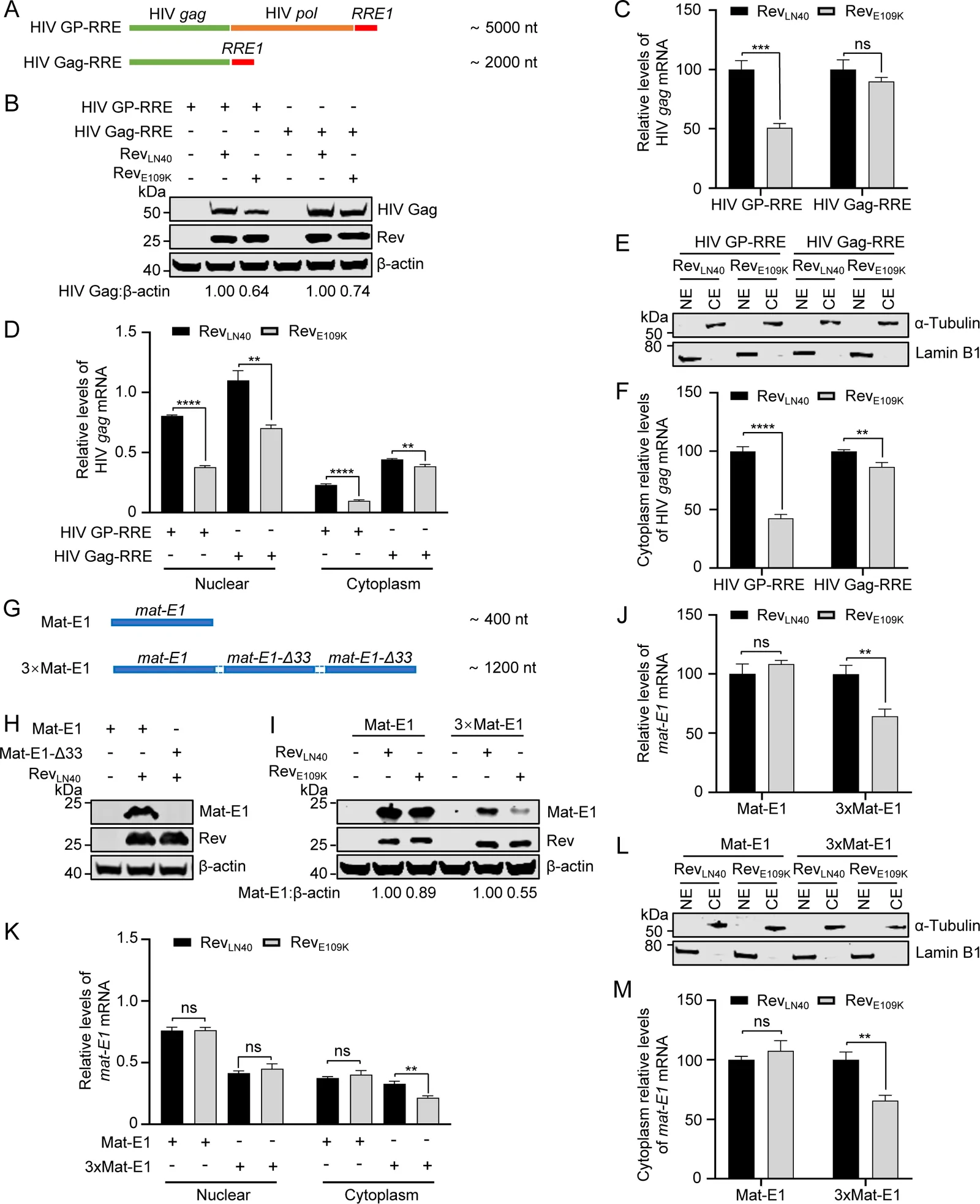
Comparison of the nuclear export activity of Rev_LN40_ and Rev_E109K_ in different RRE contexts. (**A**) Schematic diagram of two plasmids transcribed to produce HIV mRNA species with different lengths. HIV GP-RRE contains HIV *gag-pol-*coding sequences and an intact EIAV RRE1 that generates approximately 5,000 nt of HIV mRNA. HIV Gag-RRE contains HIV *gag*-coding sequences and an intact EIAV RRE1 that generates approximately 2,000 nt of HIV mRNA. (**B–F**) The nuclear export of the longer HIV *gag-pol* mRNA is more dependent on the multimerization of Rev than that of the shorter HIV *gag* mRNA. WB (**B**) and RT-qPCR (**C–F**) assays were performed to detect nuclear export and expression of the two HIV mRNA species with different lengths mediated by Rev_LN40_ or Rev_E109K_, as described in Fig. 5B through F. Band intensities of HIV Gag were determined with Odyssey CLx Image Studio and normalized to β-actin. Values were then normalized to control cells, with the control set as 1. (**G**) Schematic diagram of two plasmids transcribed to produce *mat-E1* mRNA species with different lengths. Mat-E1 contains an intact RRE2, producing approximately 400 nt of *mat-E1* mRNA. 3×Mat-E1 contains an intact RRE2 and two RRE2-deficient Mat-E1, producing approximately 1,200 nt of 3×*mat-E1* mRNA. (**H**) Mat-E1-Δ33 has no RRE2 activity. HEK293T cells were cotransfected with the Mat-E1 or Mat-E1-Δ33 construct together with the Rev_LN40_ vector or an empty vector VR1012 (as a negative control). The cells were collected at 48 hpt and were lysed for the detection of Mat-E1 and Rev expression. (**I–M**) The nuclear export of the longer 3×*mat-E1* mRNA is more dependent on the multimerization of Rev than that of the shorter *mat-E1* mRNA. WB (**I**) and RT-qPCR (**J–M**) assays were performed to detect nuclear export and expression of the two *mat-E1* mRNA species with different lengths mediated by Rev_LN40_ or Rev_E109/K_, as described in Fig. 5B through F. Band intensities of Mat-E1 were determined with Odyssey CLx Image Studio and normalized to β-actin. Values were then normalized to control cells, with the control set as 1. Error bars represent standard deviations from three independent experiments. ****, *P* < 0.0001; ***, *P* < 0.001; **, *P* < 0.01; ns, not significant (*P* > 0.05). All the experiments were performed three times, and a representative result is shown.

We note that the nuclear export of *mat-E1* and the other two mRNAs (*gag-pol* and *gag* mRNA) use different RREs in addition to their different lengths. To exclude the possibility that differences between RRE1 and RRE2 result in a specific dependence of the nuclear export of these three mRNA species on Rev_LN40_ and Rev_E109K_, we then compared the activity of Rev_LN40_ and Rev_E109K_ using the same RRE context to mediate the nuclear export of mRNAs of different lengths. First, we prepared two constructs in which the HIV *gag-pol* ORF or HIV *gag* ORF were fused with EIAV RRE1 to transcribe two HIV mRNA species with different lengths (Fig. 6A). WB (Fig. 6B) and qPCR (Fig. 6C) assays showed that there was no difference in the activity of Rev_LN40_ and Rev_E109K_ in mediating the expression of the HIV *gag* mRNA, while the activity of Rev_E109K_ was significantly less than that of Rev_LN40_ in mediating the expression of the HIV *gag-pol* mRNA, similar to the findings with EIAV mRNA. Although the accumulation of HIV *gag* mRNA in the cytoplasm mediated by Rev_E109K_ was lower than that mediated by Rev_LN40_, the difference in cytoplasmic accumulation of HIV *gag-pol* mRNA mediated by the two proteins was much greater (Fig. 6D through F). Taken together, the data shown in Fig. 5 and 6 suggest that, compared to Rev_LN40_, Rev_E109K_ mediates the nuclear export of medium-length mRNAs (EIAV and HIV *gag* mRNA) more efficiently than the nuclear export of long-length mRNAs (EIAV and HIV *gag-pol* mRNA) via RRE1. Next, we artificially generated a construct that transcribed a “3×*mat-E1*” mRNA by fusing two repeat RRE2-deficient *mat-E1* sequences to the C-terminus of wild-type *mat-E1* (Fig. 6G and H), effectively tripling the mRNA length without altering the protein product, to assess the dependence of RRE2-mediated nuclear export of mRNA of different lengths on Rev_LN40_ and Rev_E109K_. We observed that the activity of Rev_E109K_ was significantly less than that of Rev_LN40_ in mediating the expression of the “3×*mat-E1*” mRNA, while there was no difference in the activity of Rev_LN40_ and Rev_E109K_ in mediating the expression of the *mat-E1* mRNA (Fig. 6I and J). The accumulation of “3×*mat-E1*” mRNA in the cytoplasm mediated by Rev_E109K_ was lower than that mediated by Rev_LN40_, while there were no differences in the cytoplasmic accumulation of *mat-E1* mRNA mediated by these two proteins (Fig. 6K through M). Because the same 3×*mat-E1* transcript was compared between the Rev_LN40_ and Rev_E109K_ conditions, these data are more consistent with reduced Rev-mediated nuclear export efficiency of Rev_E109K_ than with intrinsic instability of the 3*×mat-E1* transcript.

The above results indicate that Rev_E109K_ mediates nuclear export of shorter mRNAs more efficiently than nuclear export of longer mRNAs regardless of whether RRE1 or RRE2 is utilized.

### Nuclear export activity of an artificial multimerization-deficient Rev is dependent on mRNA length

**Fig 7:**
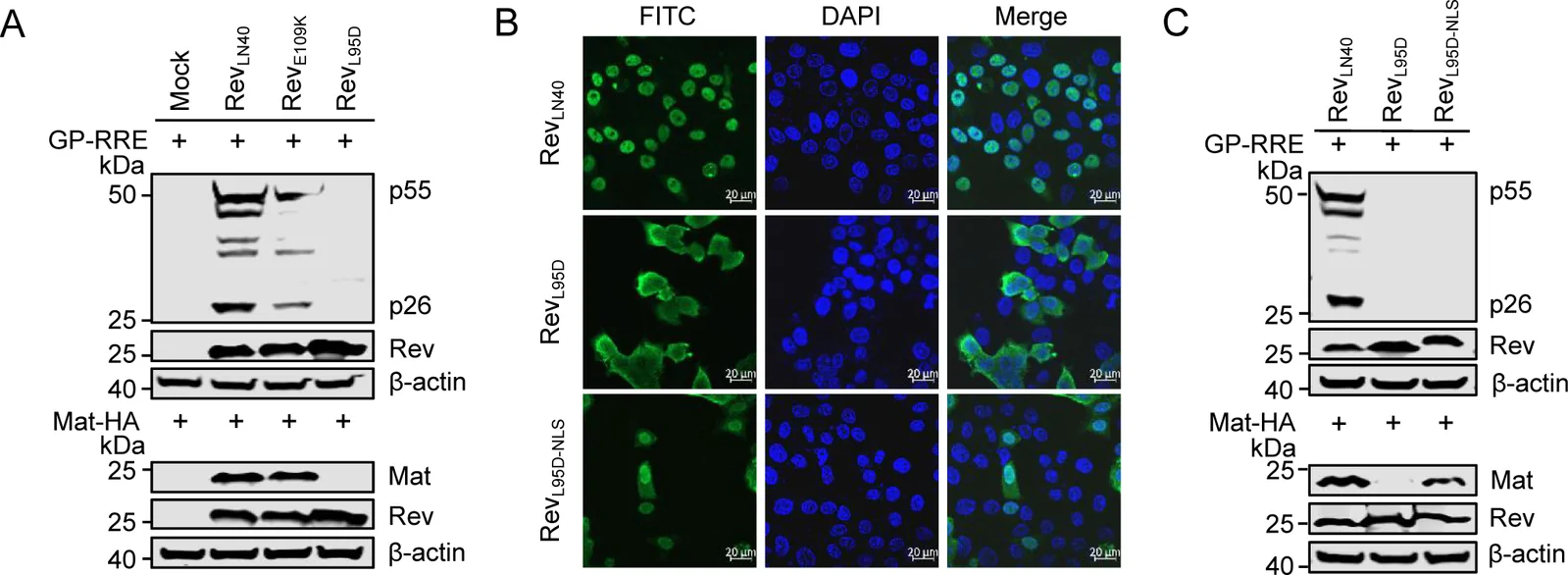
Disruption of the nuclear localization of Rev_L95D_ is the main reason for the loss of its activity in mediating Mat expression. (**A**) The effect of the L95D mutation on Rev activity. HEK293T cells were cotransfected with pGP-RRE (top) or the Mat construct (bottom) and a series of Rev expression plasmids as indicated or an empty vector VR1012 (as a negative control, Mock). The cells were collected at 48 hpt and were lysed for the detection of Gag, Mat, and Rev expression. (**B**) The L95D mutation in Rev abolished the nuclear localization of Rev. HeLa cells were separately transfected with the indicated Rev expression plasmids. At 24 hpt, the cells were fixed and stained with an anti-FLAG antibody (green), and the nuclei were stained with DAPI (blue). The subcellular localization of Rev was visualized with laser confocal microscopy. Rev_L95D_ introduces an L to D mutation at amino acid position 95 (corresponding to the EIAV_Wyoming_ strain) based on Rev_LN40_. Rev_L95D-NLS_ is based on Rev_L95D_ with the SV40 nuclear localization signal inserted at the C-terminus of the Rev-coding region. (**C**) Disruption of Rev nuclear localization resulting from the L95D mutation interferes with its activity in mediating Mat expression. HEK293T cells were cotransfected with pGP-RRE (top) or the Mat construct (bottom) and the indicated plasmids, and WB assays were performed as described for panel A.

**Fig 8:**
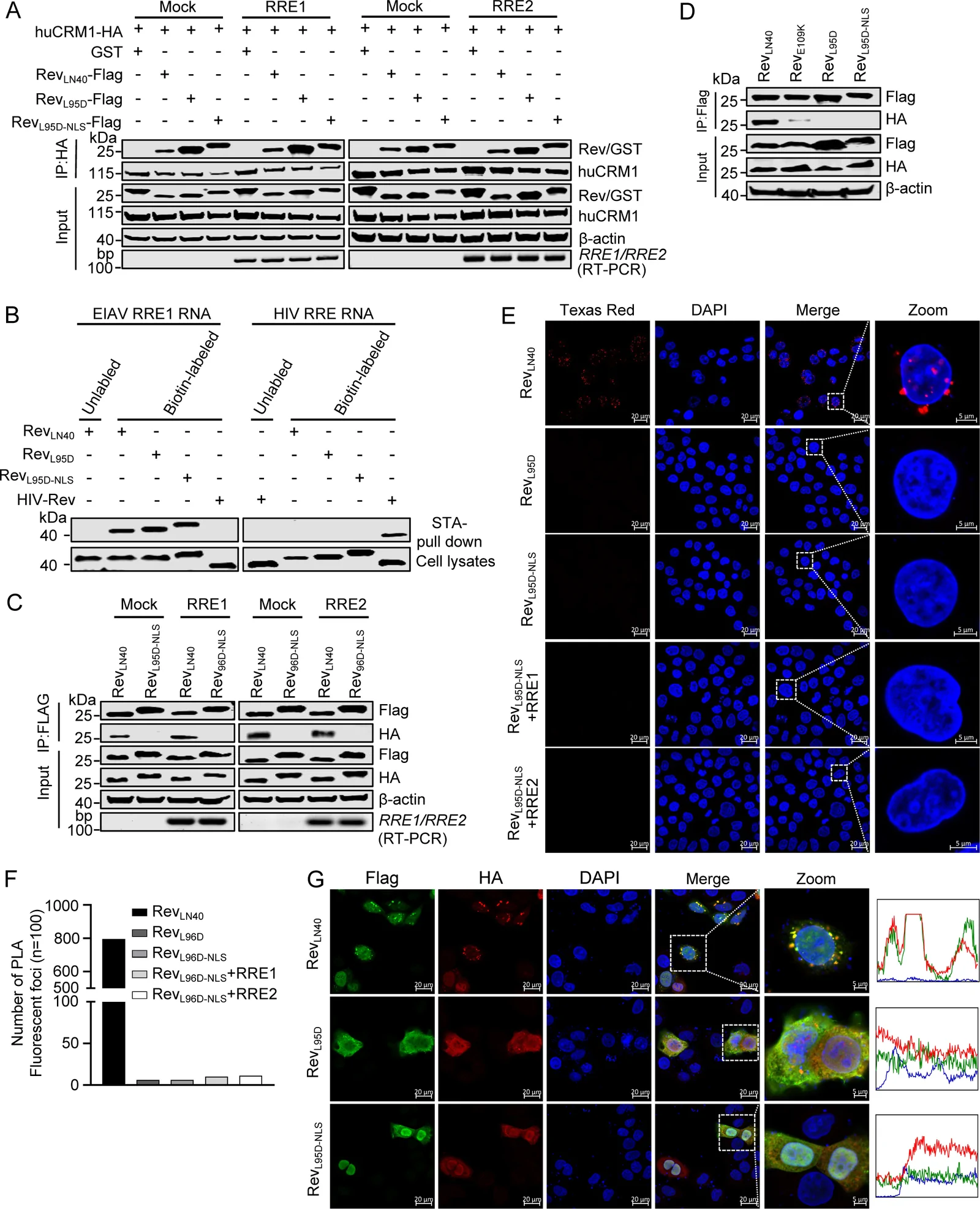
Generation of an artificial multimerization-deficient Rev mutant, Rev_L95D-NLS_. (**A**) The L95D mutation did not affect the interaction of Rev with CRM1. The Co-IP assay was performed as described in Fig. 3D, except that HA magnetic beads were used. (**B**) The L95D mutation did not affect the binding of Rev to RRE1. RNA pull-down assay was performed as described in Fig. 3F. (**C–G**) The L95D mutation disrupted Rev-Rev interactions, as assessed by Co-IP (**C and D**), PLA (**E and F**), and IFA (**G**) as described in Fig. 3G through J.

Previous studies have reported that the L95D mutation in Rev (corresponding to the EIAV_Wyoming_ strain) impairs its multimerization and, thus, disrupts its activity (30, 31). We confirmed in the context of Rev_LN40_ that the L95D mutation (Rev_L95D_) causes a loss of its activity in mediating Gag-Pol expression (Fig. 7A, top), but the mutation also causes a loss of its activity in mediating Mat expression (Fig. 7A, bottom). Surprisingly, we observed that the L95D mutation disrupts the nuclear localization of Rev (Fig. 7B). When an SV40 nuclear localization signal was added to the C-terminus of Rev_L95D_ (Rev_L95D-NLS_), its nuclear localization was partially restored (Fig. 7B), and its activity to mediate Mat expression was recovered, but its activity to mediate Gag-Pol expression was not (Fig. 7C). This result suggests that disruption of the nuclear localization of Rev_L95D_ is the main reason for the loss of its activity in mediating Mat expression. We also showed that the L95D mutation does not affect the interactions between Rev and CRM1 (Fig. 8A) or binding of Rev to the RRE1 (Fig. 8B). Notably, Co-IP analysis showed that neither Rev_L95D_ nor Rev_L95D-NLS_ was capable of self-association (Fig. 8C), confirming that the L95D mutation disrupts the Rev multimerization. We found that Rev multimerization was significantly lower in cells transfected with either Rev_L95D_ or Rev_L95D-NLS_ homologous plasmid pairs than in cells transfected with Rev_E109K_ (Fig. 8D), suggesting that the L95D mutation is much more disruptive to Rev multimerization than the E109K mutation. In addition, we also performed PLA and confirmed that the multimerization of Rev_L95D_ and Rev_L95D-NLS_ was disrupted (Fig. 8E and F). Furthermore, IFA showed that the L95D mutation abolished co-localization of FLAG- and HA-tagged Rev proteins (Fig. 8G).

**Fig 9:**
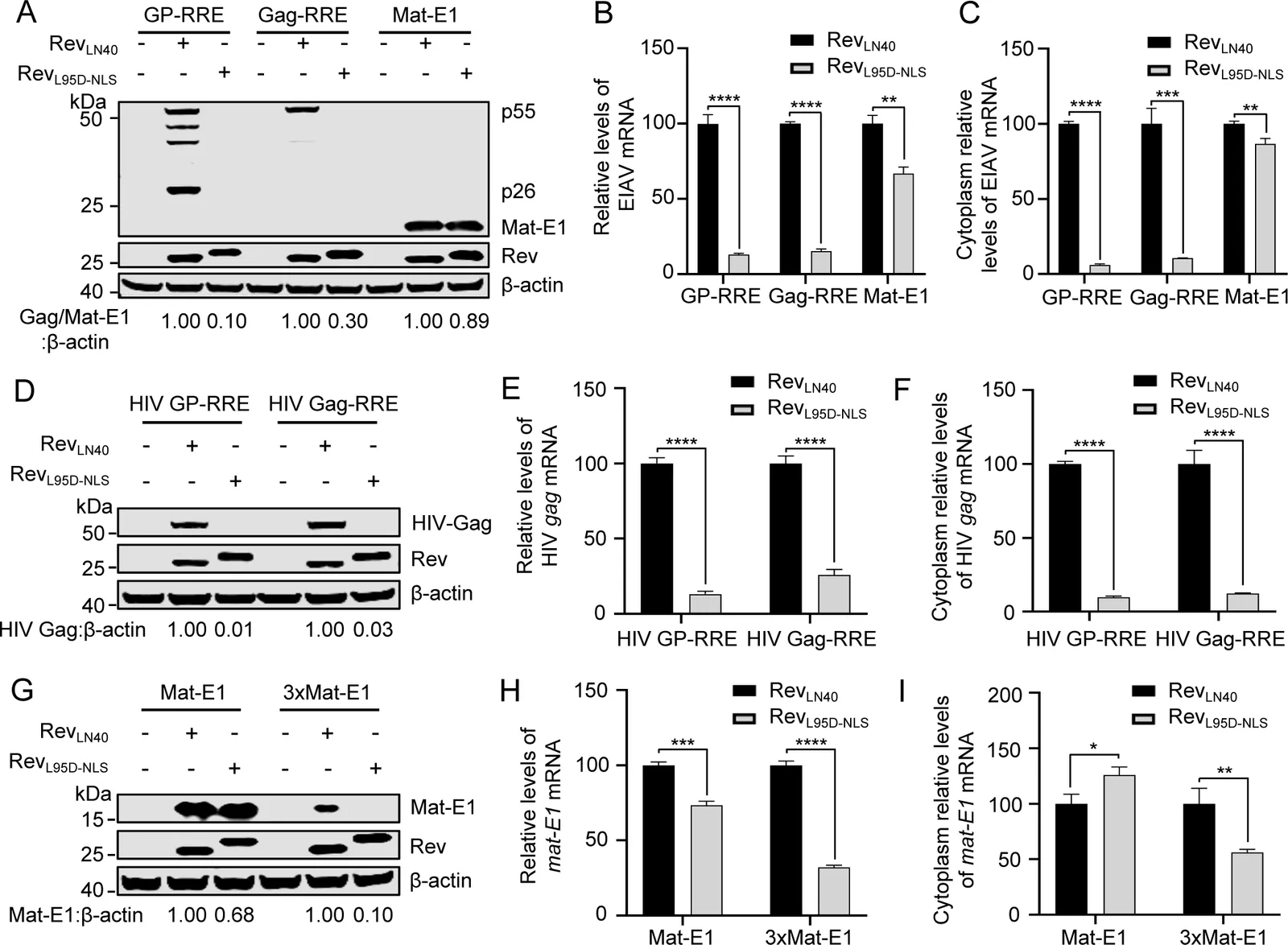
Comparison of the nuclear export efficiency of distinct mRNA species with different lengths mediated by Rev_L95D-NLS_. WB (**A, D, and G**) and RT-qPCR (**B, C, E, F, H, and I**) assays were performed to compare expression and nuclear export of the mRNA species with different lengths mediated by Rev_L95D-NLS_ and Rev_LN40_, as described in Fig. 5B through D. Error bars represent standard deviations. ****, *P* < 0.0001; ***, *P* < 0.001; **, *P* < 0.01; *, *P* < 0.05.

We then further investigated the dependence of EIAV mRNA nuclear export on Rev multimerization by using Rev_L95D-NLS_ as a second Rev multimerization-deficient mutant in addition to Rev_E109K_. As shown in Fig. 9A through I and Fig. S1A through F, the activity of Rev_L95D-NLS_ was significantly lower than that of Rev_LN40_ in mediating the expression and nuclear export of the indicated mRNAs. However, the differences in their activities decreased as the mRNA length decreased, regardless of whether the mRNAs were RRE1 or RRE2 dependent.

Taken together, these results suggest that the length of EIAV mRNA is a critical determinant of its dependence on Rev multimerization for nuclear export.

## DISCUSSION

**Fig 10:**
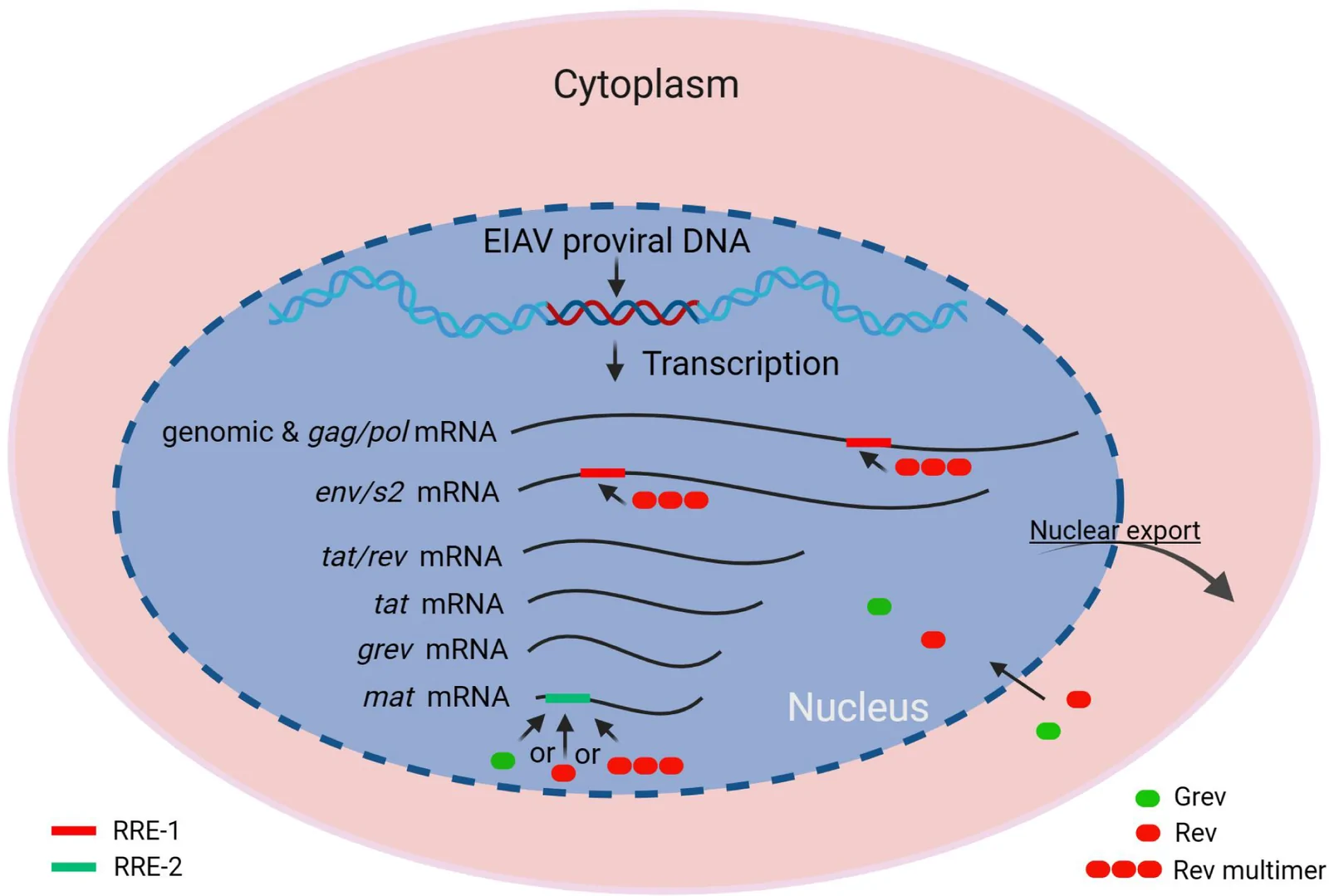
Schematic diagram of the EIAV genome transcription pattern. Six mRNA species are generated from EIAV proviral DNA and are responsible for the expression of eight viral proteins. Nuclear export and expression of full-length mRNA encoding Gag/Pol and incompletely spliced mRNA encoding Env/S2 are mediated by the Rev multimer (three red ellipses) binding to RRE1 (red line) located in the *env*-coding region. Nuclear export of mRNAs encoding Tat, Rev, or Grev uses an endogenous cellular pathway. Nuclear export and expression of mRNA encoding Mat are mediated by Rev (red ellipses) or Grev (green ellipses) via binding to RRE2 (green line), which is located in the *gag*-coding region and which overlaps with the first exon of Mat. Rev multimerization is required for Rev-mediated nuclear export of full-length or incompletely spliced mRNA but is dispensable for Rev-mediated nuclear export of *mat* mRNA.

Lentiviruses are a special class of RNA viruses that integrate into the host genome as proviral DNA. They are transcribed in the nucleus to produce viral genomic mRNA and mRNA encoding various viral proteins, which are transported to the cytoplasm through two nuclear export pathways to enable viral protein expression and viral particle assembly. The nuclear export of fully spliced lentiviral mRNAs is mediated by a host endogenous pathway (Tap/NXF1 pathway), which is also involved in the export of host mRNA. However, the nuclear export of unspliced or incompletely spliced transcripts is mediated by the Rev-CRM1 pathway (55). We recently identified a never-before-reported fully spliced transcript (designated *mat*) of EIAV that encodes a viral protein of unknown function, Mat (32). Interestingly, nuclear export of this transcript is also dependent on the Rev-CRM1 pathway. Therefore, EIAV Rev mediates not only the expression of the viral structural protein Gag-Pol but also the expression of Mat. Although Rev-mediated nuclear export of lentiviral mRNA has been well described in previous studies (8, 9, 15), the precise molecular mechanism is still incompletely understood. In the present study, we found that disruption of EIAV Rev multimerization attenuated its ability to mediate Gag-Pol expression, but not Mat expression. Our data showed that the nuclear export of two EIAV mRNA species with different lengths, a long-length species, *gag-pol* mRNA, and a short-length species, *mat* mRNA, had different tolerance to the disruption of Rev multimerization. This observation suggests that EIAV Rev multimerization may not be necessary for the Rev-mediated nuclear export of *mat* mRNA (Fig. 10).

Lentiviruses use complex mechanisms to regulate viral replication. The regulatory protein Rev mediates nuclear export of viral mRNA, an important mechanism for lentiviral replication. Therefore, Rev is absolutely required for the replication cycle of lentiviruses. However, Rev-attenuated variants are always detected during the asymptomatic phase of lentiviral disease (42, 44–46). Belshan et al. found that EIAV Rev variants isolated from the inapparent stage had significantly lower activity than those isolated from the febrile periods (42). Interestingly, attenuated Rev activity, reduced Gag expression, and increased resistance to CTL killing were observed in HIV-1 variants from asymptomatic patients (45, 46), suggesting that diminished Rev activity may escape host immune killing by downregulating the expression of structural proteins such as Gag. Thus, Rev may be an important factor influencing the transition from latent to active status during lentiviral infection (13). Lentiviruses, including EIAV, exist as complex quasispecies (35, 38, 40). In the present study, a rare Rev variant with an E109K mutation was identified in the bone marrow of EIAV-infected horses. This variant mediated significantly reduced activity of Gag-Pol expression, leading us to conclude that this mutant may be derived from a virus quasispecies in a latently infected state.

We note that the E109K mutation in Rev is caused by a mutation from G to A in the nucleotide sequence, or more precisely, a mutation from codon GAA (E) to AAA (K). The host restriction factor APOBEC3 (A3) is known to cause G-to-A hypermutations in a variety of retroviruses (56–60). Previous studies found that equine A3 is expressed in several tissues including bone marrow, lymph nodes, and spleen and demonstrated that it can strongly inhibit EIAV replication by introducing G-A hypermutation (61, 62). A3-mediated G-to-A hypermutations have a strong dinucleotide preference (60, 63). Bogerd et al. showed that the equine A3-mediated G-to-A hypermutation also has a dinucleotide preference of GG to AG and GA to AA (62). Therefore, it is possible that the E109K mutation is a result of the action of A3 protein.

In lentiviruses, the *gag* and *pol* genes partially overlap and are on different reading frames. Both genes encode products that are generated from full-length genomic viral mRNA. Translation of the *gag* and *pol* genes is regulated by a ribosomal frameshifting event to produce a Gag polyprotein and a Gag-Pol polyprotein (64, 65), which are produced at a ratio of approximately 20:1 (66). Both polyproteins are cleaved by the viral protease (PR) produced as part of Gag-Pol. The EIAV Gag polyprotein precursor (p55) is cleaved by PR into four major internal structural proteins of the mature virion, including the matrix (MA, p15), the capsid (CA, p26), the nucleocapsid (NC, p11), and p9. p55 and p26, as well as several other intermediate cleavage products, are easily detected from EIAV-infected or transfected cell lysates using mAbs against p26 in WB assays (52, 67). In the present study, the levels of Gag expression were used to indicate the activity of Rev in mediating Gag-Pol expression. However, we observed that in the Rev_E109K_-mediated Gag-Pol expression experiments, the reduction of p26 and other intermediates was more severe than the reduction of p55 in most cases (Fig. 1B through D, 4A, and 5B), which may be due to the reduction of Pol expression resulting in less efficient processing of the Gag precursor.

Numerous studies have demonstrated that Rev multimerization is an important step in structural gene expression and lentiviral replication (13, 14, 18). HIV-1 Rev contains two separate multimerization functional domains (15). Umunnakwe et al. reported that a coiled-coil motif consisting of 29 amino acids in the central region of EIAV Rev is responsible for its multimerization (31). In the present study, the E109K mutation is located in this coiled-coil motif (Fig. 2A), and we confirmed that the mutation disrupts the multimerization of Rev using the self-association assay, which is commonly used to study protein multimerization (68, 69). This result also supports the findings of Umunnakwe et al. (31). Furthermore, the mutation did not affect the nuclear localization, the RRE binding, or the interaction of Rev with CRM1. We observed that the 109 position of Rev (corresponding to the EIAV_LN40_ strain used in this study) was variable among EIAV strains, and the changes did not affect Rev activity (Fig. 2), suggesting that these published Rev sequences may have been derived from active EIAV isolates. In addition, we found that the properties of the amino acid at the 109 site were not a major factor affecting Rev activity, and that only the presence of K at this site caused an attenuation of Rev activity in mediating Gag-Pol expression. This could be a result of K inducing a certain kind of chemical modification or leading to a drastic change in the spatial structure of Rev.

An L95D mutation in EIAV Rev, which has been reported in previous studies to impair multimerization and, thus, lead to a reduction in Rev activity (31, 70), was also observed to abolish nuclear localization of Rev in the present study (Fig. 7B). Furthermore, fusing the SV40 nuclear localization signal to Rev rescued the disruption of Rev nuclear localization by the L95D mutation (Fig. 7B), but not the disruption of Rev multimerization (Fig. 8C through G). The classical nuclear localization signal (NLS) is rich in arginine (R) or lysine (K) residues. Previous studies have shown that the five basic amino acids (KRRRK) at the C-terminus of EIAV Rev are its NLS, and substitution of any two adjacent basic residues in the basic motif with alanine (A) disrupts its nuclear localization (29, 30). It has been reported that NLS of BIV Rev is bipartite, harboring a 20 aa spacer sequence between the motifs that compose the NLS (71). Therefore, we speculate that EIAV Rev may have a special NLS (72), which is bipartite and contains a spacer sequence of more than 60 amino acids (aa 95–158). Of course, this needs to be verified in the future.

Previous studies have shown that a threshold level of Rev is required for effective HIV-1 replication (73), suggesting that Rev must accumulate to a certain concentration and form multimers to trigger export and expression of viral mRNAs encoding structural gene products. However, we observed that disruption of EIAV Rev multimerization did not attenuate its activity in mediating Mat expression. Interestingly, at low Rev concentrations, the activity of Rev_E109K_ in mediating Mat expression is stronger than Rev_LN40_ (Fig. 1G). This suggests that Mat expression may be independent of Rev multimerization, or that Rev activity may be dispensable for its own multimerization when targeting *mat* mRNAs. A previous study reported that excessive Rev can exert an inhibitory effect on its own nuclear export activity (74), and this concentration-dependent activity pattern further supports a potential regulatory role of Rev expression levels in its functional performance. In addition, we recently found that Grev, another EIAV-encoded post-transcriptional regulator that is not detected in a multimeric form in cells, also mediates Mat expression in a CRM1-dependent pattern (75). These data raise the possibility that Mat expression may occur earlier than Gag-Pol expression, potentially during a phase when Rev levels are relatively low and below the threshold required for efficient export of long viral transcripts. This temporally regulated expression model may represent a mechanism that allows EIAV to produce Mat before robust expression of structural proteins; however, the precise timing of Mat expression and its biological function remain to be determined. This possibility is consistent with previous studies investigating the kinetics of HIV-1 RNA expression during early infection (76). In HIV-1 infection, env-specific and rev-specific singly spliced mRNAs (shorter transcripts) were detected at early stages, whereas unspliced full-length genomic RNA (a longer transcript) was not readily detected during the initial phase of infection. Furthermore, another study reported that Env expression from singly spliced transcripts requires lower levels of Rev than Gag-Pol expression from full-length unspliced genomic RNA (77). Together, these observations support the possibility that shorter viral transcripts may have distinct requirements for Rev activity.

Cis-acting repressing sequence (CRS) is a type of RNA sequence element within cellular or viral mRNAs that controls gene expression by inhibiting nuclear export of the mRNA through binding of a variety of host proteins (78). Previous studies have identified the presence of multiple CRSs in the Gag-, Pol-, and Env-coding regions of HIV-1 and EIAV (79, 80). Moreover, it has been demonstrated that these CRSs inhibit nuclear export of viral mRNAs, but that this inhibitory effect can be neutralized by the supply of Rev (79–81). In our study, the EIAV *gag-pol* and *mat* gene constructs exhibited poor expression and cytoplasmic RNA accumulation in the absence of Rev, which may, therefore, be due mainly to the inhibitory effect of the potential CRS within them. We also found that disruption of Rev multimerization caused by the E109K mutation strongly attenuated its activity to mediate Gag-Pol expression, but not Mat expression (Fig. 1B through G, Fig. 4A and B, and Fig. 5B), demonstrating that Rev-mediated nuclear export of *gag-pol* mRNA is distinct from that of *mat* mRNA, and that the latter is not strictly dependent on Rev multimerization.

Previous studies have shown that RNA length is an important determinant of RNA export pathway selection (53, 54). In fact, the EIAV *gag-pol* mRNA and *mat* mRNA species used in our experiments have different lengths: the former being more than 5,000 nt and the latter less than 400 nt. Moreover, we also demonstrated that the difference between Rev multimerization-deficient mutants (Rev_E109K_ and Rev_L95D-NLS_) and wild-type Rev in their ability to mediate the nuclear export of mRNAs decreases as the mRNAs become progressively shorter (Fig. 5, 6, and 9). We hypothesized that since the long-length species *gag-pol* mRNA contains more CRS than the short-length species *mat* mRNA, its nuclear export resistance is greater and, therefore, more Rev is required to drive its nuclear export. Alternatively, although both Rev-mediated Mat expression and Rev-mediated Gag-Pol expression are dependent on CRM1 (32), Rev-mediated nuclear export of mRNAs also requires the recruitment of a variety of host proteins in addition to CRM1 (82). Disruption of Rev multimerization may reduce the type or number of host proteins it recruits, resulting in a decrease in the “carrying capacity” of Rev. This possibility implies that both Rev-mediated *gag-pol* mRNA nuclear export and Rev-mediated *mat* mRNA nuclear export employ different adaptor proteins in a CRM1-dependent manner, which requires further investigation. This view is also supported by a previous study that showed that nuclear export of *gag-pol* mRNA and *mat* mRNA depends on different functional domains of Rev (32). Notably, in addition to their different lengths, *gag-pol* mRNA and *mat* mRNA utilize separate RREs for nuclear export, the former using RRE1 located in the Env-coding region and the latter using RRE2 located in the Gag-coding region. These two RREs are not functionally interchangeable so that the RREs may also be an important factor causing the different activity of multimerization-deficient Rev in mediating the nuclear export of *gag-pol* mRNA and *mat* mRNA. However, we excluded this possibility because we observed that the activity of multimerization-deficient Revs in mediating mRNA nuclear export decreased with increasing the mRNA length, both via RRE1-dependent manner and via RRE2-dependent manner (Fig. 5, 6, and 9). These data also highlighted a consistent length-dependent sensitivity for the activity of multimerization-deficient Revs in distinct mRNA contexts. Therefore, we concluded that the different dependence of nuclear export of *gag-pol* mRNA and *mat* mRNA on Rev multimerization is modulated by the length of the mRNA. Nevertheless, the precise molecular mechanisms underlying this length-dependent regulation remain to be fully elucidated and may involve CRS elements, RRE secondary structures, as well as additional host cofactors including RanGTP; all of these factors exert critical functions during Rev-CRM1-mediated nuclear export.

In summary, we found that EIAV Rev multimerization is absolutely required for the nuclear export of *gag-pol* mRNA, whereas it is dispensable for the nuclear export of *mat* mRNA. We further demonstrated that transcript length is strongly associated with the dependence of Rev-mediated nuclear export of mRNA on Rev multimerization. These findings deepen our understanding of the mechanism underlying Rev nuclear export activity in lentiviruses and emphasize that Rev-mediated post-transcriptional regulation in lentiviruses is diverse and complex.

## MATERIALS AND METHODS

### Amplification and cloning of the EIAV *rev* gene

EIAV mRNA was extracted from the bone marrow and lymph nodes of a horse infected with EIAV_LN40_ using the TRIzol method, as previously described (32). The complete *rev* gene cDNA fragments were amplified with reverse transcription (RT)-PCR using Kod FX Neo polymerase (Toyobo, Japan). The primer pair LN40Rev-F (5′-AAG AAG AAG AAC AAA CAA AGA CAG-3′) and LN40Rev-R (5′-AGC AGC CAT AGT GGG AAG CCA CAG-3′) were used for PCR. Three independent RT-PCR experiments were performed, and the PCR products were excised from 0.8% agarose gels and ligated into the pMD18-T vector (TaKaRa, Dalian, China). Twenty-one and eighteen positive recombinant clones were obtained from the bone marrow and lymph node samples, respectively, and were sequenced.

### Sequence alignments

All *rev* sequences obtained in this study were organized and translated into amino acid sequences using the EditSeq tool in the Lasergene DNAStar 7.1 software. Amino acid sequence alignment was performed using ClustalW.

### Plasmid construction

To construct plasmids expressing FLAG-tagged or hemagglutinin (HA)-tagged Rev, the *rev* gene sequence from the EIAV virulent strain EIAV_LN40_, or the EIAV attenuated strain EIAV_FDDV13_ was inserted into a VR1012 vector with a FLAG tag or into a pCMV vector with an HA tag, respectively. A series of expression vectors with substitution mutations in the *rev* gene was constructed based on VR-Rev-Flag or pCMV-HA-Rev using mutagenesis PCR. The following plasmids and viral systems used in this study have been described previously: the Rev-dependent EIAV Gag-Pol expression plasmid pGP-RRE; Mat expression constructs including pcDNA-Mat-HA, pcDNA-Mat-E1-HA, and pcDNA-Mat-E1; the HIV-1 Rev expression plasmid VR-Rev_HIV_-Flag; the human CRM1 expression plasmid pcDNA-huCRM1-HA; the full-length EIAV infectious clone CMV3-8; and the three-plasmid HIV-1 reporter virus system consisting of pSIN4-EF1a-IRES-GFP, psPAX2, and pMD2.G (32, 52, 75). The EIAV RRE1 sequence was amplified from the pGP-RRE plasmid, and the HIV RRE sequence was amplified from the HIV infectious clone pNL4-3 using PCR and cloned into the pcDNA3.1 vector to construct the pcDNA-RRE1_EIAV_ and pcDNA-RRE_HIV_ plasmids to assess the binding of the Rev mutant to RRE RNA. The EIAV molecular clone with the E109K mutation, CMV3-8/Rev_E109K_, was constructed based on CMV3-8 using mutagenesis PCR. The intact EIAV *gag* ORF and intact RRE1 were PCR amplified from the pGP-RRE and inserted into the pcDNA3.1 vector to construct the pGag-RRE plasmid. The FLAG-tagged Rev was inserted into the pSIN4-EF1a-IRES-GFP vector to construct the pSIN4-EF1a-Rev-Flag-IRES-GFP plasmid. The pGP_HIV_-RRE and pGag_HIV_-RRE plasmids were constructed by inserting PCR-amplified HIV elements (intact *gag-pol* mRNA or *gag* mRNA from infectious clone pNL4-3) and EIAV RRE1 (amplified from pGP-RRE) into pcDNA3.1 vector. The pcDNA-Mat-E1-Δ33 plasmid was generated using mutagenesis PCR, introducing a 33-nt deletion (corresponding to amino acids 1–11) in the Mat-E1-coding sequence. The C-terminus of pcDNA-Mat-E1-HA was fused with two repeats of mat-E1-Δ33, tripling the mRNA transcript length while preserving the original protein product. To restore the nuclear localization of Rev_L95D_, VR-Rev_L95D-NLS_-Flag and pCMVHA-Rev_L95D-NLS_ plasmids were constructed by fusing the nuclear localization signal of SV40 to the C-terminus of Rev_L95D_. All constructed plasmids were analyzed and verified by sequencing.

### Cell culture and transfection

HEK293T cells and HeLa cells were cultured in Dulbecco’s modified Eagle’s medium (DMEM)-high glucose (Sigma-Aldrich, USA) containing 10% fetal bovine serum (Sigma-Aldrich, USA) and 1% penicillin-streptomycin (Beyotime, China). Fetal donkey dermal (FDD) cell cultures were maintained in minimal essential medium (α-MEM, Biosharp, China) containing 10% fetal bovine serum (Sigma-Aldrich, USA) and 1% penicillin-streptomycin (Beyotime, China). Equine monocyte-derived macrophage (eMDM) cells were isolated from fresh heparinized horse whole blood and cultured in RPMI 1640 (Sigma-Aldrich, USA) containing 30% donor equine serum (HyClone, USA), 30% Newborn Calf Serum (Ausbian, Australia), and 1% penicillin-streptomycin (Beyotime, China) as previously described (49). All cells were cultured in a humidified incubator at 37°C with 5% CO_2_. Cells were transiently transfected with the indicated plasmids using PolyJet DNA reagent (SignaGen, Rockville, MD, USA) *in vitro*, following the manufacturer’s instructions.

### Co-immunoprecipitation assay and western blotting

Transfected cells were lysed with lysis buffer (150 mM Tris-HCl [pH 7.6], 50 mM NaCl, 5 mM EDTA, and 1% Triton X-100) and centrifuged at 8,000 rpm for 5 min at 4°C. The crude lysates were incubated with anti-FLAG or anti-HA magnetic beads under rotation at 4°C for 2 h. After incubation, the beads were collected using a magnetic separator and subsequently washed five times with PBS. The anti-FLAG magnetic beads were incubated with a 3× FLAG peptide at 4°C for 1 h to elute the bead-bound materials. For the anti-HA magnetic beads, the bead-bound complexes were directly mixed with SDS-PAGE loading buffer. All samples were then boiled at 98°C for 10 min and subjected to western blotting (WB) analysis. For WB, samples were separated using SDS-PAGE and then transferred to NC membranes (Millipore, Germany). The membranes were blocked with 5% skim milk in PBS for 2 h at room temperature. Primary antibodies were incubated overnight at 4°C, and then the membranes were washed three times for 10 min with TBST. Secondary antibodies were incubated for 1 h at room temperature in the dark, and then the membranes were washed three times for 10 min with TBST. The membranes were analyzed using the Odyssey imaging system (LI-COR, USA). Band intensities of target proteins were quantified using Odyssey CLx Image Studio software. Protein expression levels were normalized to β-actin as an internal control, and the relative intensities were further normalized to the control group, which was set to 1.

### Immunofluorescence assay

HeLa cells were seeded on glass-bottom cell culture dishes and transfected with the indicated plasmids using PolyJet DNA transfection reagent (SignaGen, USA). After 24 h, cells were fixed with 4% paraformaldehyde (Beyotime, China) for 30 min and then permeabilized with 0.1% Triton X-100 (Beyotime, China) for 10 min at room temperature. The cells were blocked with skim milk in PBS for 1 h and then labeled with an anti-FLAG antibody or an anti-HA antibody. The samples were then incubated with Alexa Fluor 488-conjugated goat anti-mouse antibody or Alexa Fluor 647-conjugated goat anti-rabbit antibody (Thermo Scientific, USA) in PBS containing 5% skim milk for 1 h at room temperature. To stain the nuclei, the cells were incubated with 2-(4-amidinophenyl)−6-indolecarbamidine dihydrochloride (DAPI) (Beyotime, China) for 10 min. Cells were observed and analyzed using a confocal microscopy (ZEISS LSM880, Thornwood, NY, USA). The histogram of fluorescent intensity from the merged image was obtained using ZEISS ZEN Blue software.

### Proximity ligation assay

PLA was performed using Duolink *In Situ* reagents (Sigma-Aldrich, USA) according to the manufacturer’s protocol. Briefly, HeLa cells were seeded on glass-bottom cell culture dishes and transfected with the indicated plasmids using PolyJet DNA transfection reagent (SignaGen, USA). After 24 h, cells were fixed and permeabilized as described in IFA. The cells were blocked with blocking buffer for 1 h and then labeled with mouse anti-FLAG antibody and rabbit anti-HA antibody. Subsequently, the samples were incubated for 1 h at 37°C with species-specific PLA probes (anti-mouse plus, anti-rabbit minus). The ligation reaction was initiated by incubating the samples at 37°C for 30 min. Subsequently, rolling-circle amplification was performed using fluorophore-labeled detection oligonucleotides for 100 min at 37°C. The samples were mounted with Duolink *In Situ* Mounting Medium containing DAPI. Cells were observed and analyzed using a confocal microscopy (ZEISS LSM880, Thornwood, NY, USA). The number of PLA foci per cell was quantified in 100 cells.

### Cell fractionation and quantitative real-time PCR

HEK293T cells were transfected with the desired plasmids. At 24 hpt, the total RNA was extracted using the simply P total RNA Extraction kit (BioFlux, USA) or the TRIzol method. The RNA from the nucleus and cytoplasm was purified using the PARIS protein and RNA isolation kit (Invitrogen, Thermo Fisher Scientific, USA) and then subjected to reverse transcription using the PrimeScript RT reagent kit with gDNA Eraser (TaKaRa, Japan) according to the manufacturer’s instructions. Total RNA was analyzed using SYBR Green (Takara, Japan)-based relative quantitative PCR (q-PCR) with the 2^−ΔΔCT^ method to determine the relative expression levels of EIAV *gag* mRNA, HIV *gag* mRNA, *mat*-E1, and 3×*mat*-E1 mRNA. Nuclear and cytoplasmic RNA fractions (except for samples in Fig. 3A) were quantified via absolute q-PCR with standard curves to measure absolute transcript copy numbers. The EIAV *gag* mRNA, HIV *gag* mRNA, *mat*-E1, and 3×*mat*-E1 mRNA expression levels were normalized to those of β-actin or GAPDH. The following primers were used for real-time RT-PCR: *gag*-forward (5′-CGA TGC CAA ATC CTC CAT TAG-3′), *gag*-reverse (5′-CTG ATC AAA AGC AGG TTC CAT CT-3′); HIV *gag*-forward (5′-AAA GGC ACA GCA AGC AGC AG-3′), HIV *gag*-reverse (5′-ACC ATT TGC CCC TGG AGG TT-3′); *mat-E1*-forward (5′-AGT TAG AGA AGG TGA CGG TAC-3′), *mat-E1*-reverse (5′-CAA CAA TGG AAT GAC GTC CCT-3′); 3×*mat-E1*-forward (5′-GAA CCC ACT GCT TAC TGG CT-3′), 3×*mat-E1*-reverse (5′-GCG CTT TGC TCC ATG TCA AA-3′); *β-actin*-forward (5′-ACG GCA TCG TCA CCA ACT G-3′), *β-actin*-reverse (5′-CAA ACA TGA TCT GGG TCA TCT TCT C-3′); *GAPDH*-forward (5′-GAC ACC CAC TCC TCC ACC TTT-3′), and *GAPDH*-reverse (5′-ACC ACC CTG TTG CTG TAG CC-3′) (83). The distribution ratio of EIAV mRNA in the nucleus and cytoplasm from the same sample was analyzed as described previously (84).

### RNA pulldown assay

The pcDNA-RRE1_EIAV_ plasmid or pcDNA-RRE_HIV_ plasmid was linearized by digestion with the restriction endonuclease *Not* I (Thermo Scientific, USA), and RRE RNA was transcriptionally amplified *in vitro* using the RiboMAX Large Scale RNA Production System (Promega, USA). The T4 RNA ligase was then used to attach a single biotinylated nucleotide to the 3′ terminus of an RNA strand (Thermo Scientific, USA). HEK293T cells were subsequently transfected with the indicated plasmids. At 48 hpt, the transfected cells were lysed with lysis buffer and centrifuged at 8,000 rpm for 5 min at 4°C. The lysate was incubated with 50 pmol of biotin-labeled RNA at 4°C overnight and then with 50 μL of streptavidin affinity magnetic beads for 1 h at 4°C. The magnetic beads were washed with washing buffer two times. The magnetic beads were incubated with Elution Buffer for 30 min to elute the bead-bound materials. A WB assay was then performed to assess the binding of the RNA to the protein.

### Quantification of EIAV viral titer with the RT activity assay

EIAV viral titers were quantified using reverse transcriptase (RT) activity kits (colorimetric kits, Roche, Basel, Switzerland) according to the manufacturer’s instructions. The RT activity was expressed as optical density (OD) values and was determined from the absorbance at 405 nm. Finally, EIAV viral titers were calculated based on the standard curve.

### Viral growth kinetics

eMDMs were infected with an equal amount (RT content) of either EIAV_CMV3-8_ or EIAV_CMV3-8/RevE109K_, and the supernatant was replaced with fresh medium at 2 hpi. Equal volumes of cell culture supernatants were collected at the indicated time points. Viral RNA was extracted from the cell supernatants with the QIAamp Viral RNA Mini Kit (Qiagen, Hilden, Germany) and then reverse transcribed using the PrimeScript RT reagent kit with gDNA Eraser (TaKaRa, Japan). Viral growth kinetics were determined using a qPCR assay of EIAV *gag* mRNA as described previously (48).

### Production of pseudotyped HIV encoding EIAV Rev_LN40_

HEK293T cells were co-transfected with lentiviral packaging plasmids psPAX2, pMD2.G, and the corresponding transfer plasmid (pSIN4-EF1a-Rev-Flag-IRES-GFP or empty control pSIN4-EF1a-IRES-GFP) using PolyJet DNA reagent. At 48 h post-transfection, viral supernatants were harvested, clarified by centrifugation at 1,000 × *g* for 10 min followed by filtration through a 0.45-μm filter to remove cell debris, and then concentrated by ultracentrifugation at 20,000 × *g*, 4°C for 2 h. The resulting viral pellets were resuspended in Opti-MEM medium and used for infection of eMDMs.

### Semi-quantitative RT-PCR assay for comparison of EIAV gene expression

HEK293T cells were transfected with the desired plasmids. At 24 hpt, total RNA was extracted using a TRIzol method, followed by reverse transcription using the PrimeScript RT Reagent Kit with gDNA Eraser (TaKaRa, Japan) according to the manufacturer’s directions. PCR was performed using Kod FX Neo polymerase (Toyobo, Japan) and the following protocol: 2 min at 98°C; 30 cycles of 30 s at 98°C, 30 s at 56°C, and 20 s at 68°C; and 10 min at 68°C. Nested-PCRs were performed under similar conditions. The primer pair *gag*-forward (5′-CGA TGC CAA ATC CTC CAT TAG-3′) and *gag*-reverse (5′-CTG ATC AAA AGC AGG TTC CAT CT-3′) were used to amplify *gag*-specific transcripts. The nested primer pairs *mat*-p555-forward (5′-TCA AAA GCT AAC TAA TGG TAA C-3′)/*mat*-p7878-reverse (5′-ATT GAG GCA TTG TTA CAT GAG A-3′) and *mat*-p643-forward (5′-CAA TTA AGG GAC GTC ATT CCA T-3′) /*mat*-p7855-reverse (5′-GTA GCT GGA TTT AGC ATT TTC C-3′) were used to amplify *mat* specific transcripts. β-actin-specific transcripts were amplified using the primer pair *β-actin*-forward (5′-ACG GCA TCG TCA CCA ACT G-3′) and *β-actin*-reverse (5′-CAA ACA TGA TCT GGG TCA TCT TCT C-3′). RRE1-specific transcripts were amplified using the primer pair *RRE1*-forward (5′-TCC CAG GGG GAA TAT CAA CC-3′) and *RRE1*-reverse (5′-CCC CTC ATA CCA CCA GAG GA-3′). RRE2-specific transcripts were amplified using the primer pair *RRE2*-forward (5′-CAT GGA GCA AAG CGC TCA AG-3′) and *RRE2*-reverse (5′-TCT GGG AAA CGT CCT CCA AC-3′). PCR products were fractionated by electrophoresis in 0.8% agarose gels containing ethidium bromide.

## Data Availability

The EIAV *rev* sequences generated in this study are available in NCBI under accession numbers PZ962335 to PZ962373.

## SUPPLEMENTAL MATERIAL

Supplemental Material mbio.00841-26-s0001.pdf Figure S1.

ASM does not own the copyrights to Supplemental Material that may be linked to, or accessed through, an article. The authors have granted ASM a non-exclusive, world-wide license to publish the Supplemental Material files. Please contact the corresponding author directly for reuse.
