# Supplementary material for: The dependence of Rev-mediated nuclear export of EIAV mRNA on Rev multimerization is modulated by mRNA length
Source: mBio. 2026 Oct 9;17(11):e00841-26. doi: 10.1128/mbio.00841-26 (PMC13753803; doi:10.1128/mbio.00841-26)
Supplement: Supplementary file 1 [file mbio.00841-26-s0001.pdf]

The dependence of Rev-mediated nuclear export of EIAV mRNA on Rev multimerization is modulated by mRNA length

Weiwei Ma[1], Mengmeng Zhang[1], Xiangmin Zhang[1], Liuke Sun[1], Jiwei Li[1], Yang Zhao[1], Xiaohua Ma[1], Weiguo Zhang[1], Bowen Bai[1], Xue-Feng Wang[1]*, Xiaojun Wang[1,2,3]*

[1] State Key Laboratory of Animal Disease Control and Prevention, Harbin Veterinary Research Institute of Chinese Academy of Agricultural Sciences, Harbin 150069, China

[2]Institute of Western Agriculture, the Chinese Academy of Agricultural sciences, Changji 831100, PR China

[3]China-Kazakhstan Joint Laboratory for Herbivorous Animal Disease Research, Heilongjiang Province， Harbin Veterinary Research Institute,Chinese Academy of Agricultural Sciences, Harbin 150069, China

*Corresponding Author.

E-mail: wangxiaojun@caas.cn (XW)； wangxuefeng@caas.cn (X-F.W)

Supplemental Material

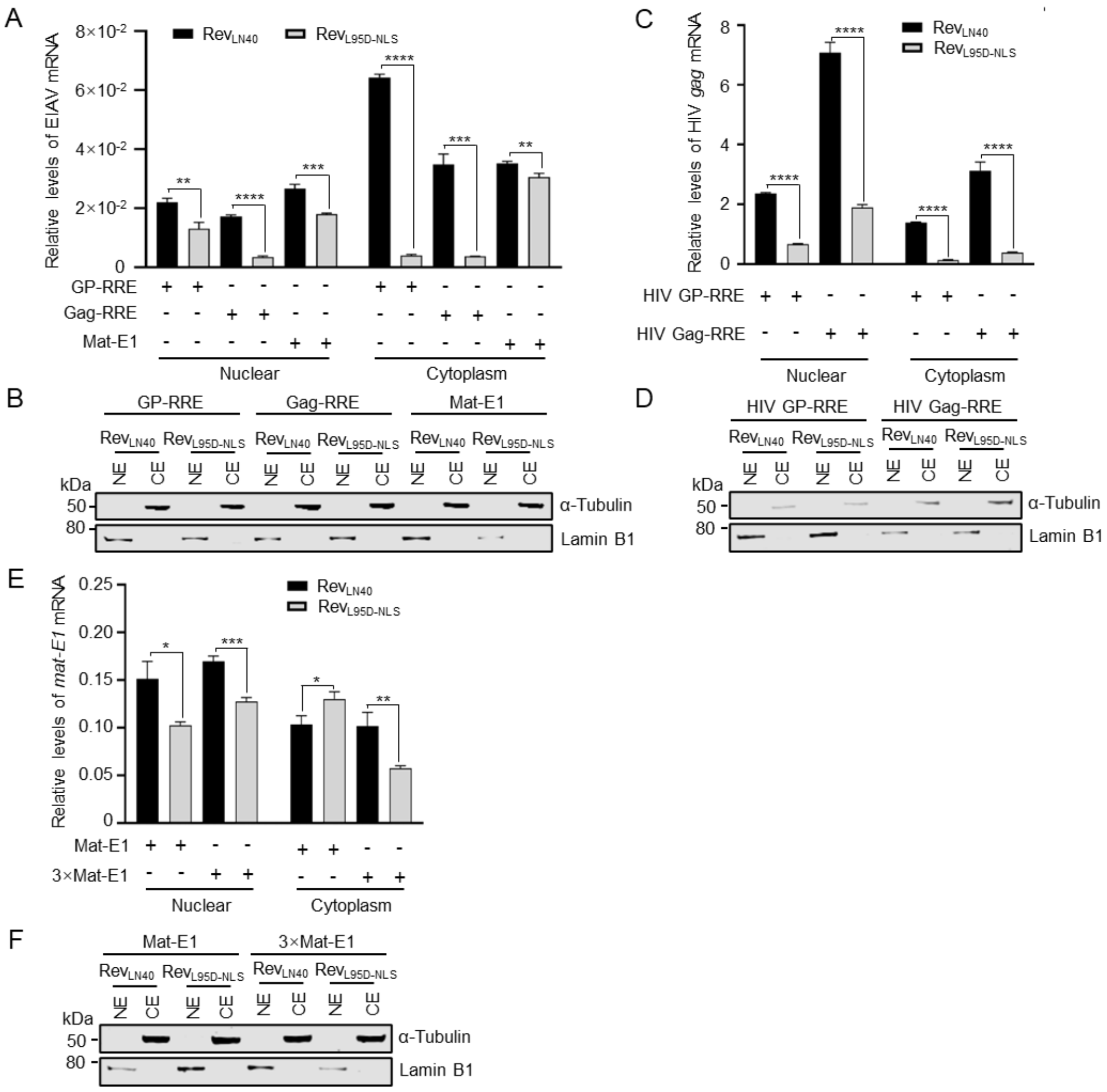

Figure S1. Nuclear and cytoplasmic distribution of different mRNA species with different lengths in the presence of Rev$_{L95D\text{-}NLS}$. HEK293T cells were cotransfected a series of plasmids as indicated. The nuclear and cytoplasmic RNA fractions were extracted at 24 hpt. Levels of mRNA in cytoplasmic and nuclear fractions were determined using RT-qPCR (A, C and E). GAPDH were measured as an internal reference. Error bars represent standard deviations of three independent experiments. ****, $p < 0.0001$; ***, $p < 0.001$; **, $p < 0.01$; *, $p < 0.05$. WB of Lamin B1 and α-Tubulin was conducted to assess the effectiveness of cell fractionation (B, D and F). All the experiments were performed three times, and a representative result is shown.
